# On Practical Issues for Stochastic STDP Hardware With 1-bit Synaptic Weights

**DOI:** 10.3389/fnins.2018.00665

**Published:** 2018-10-15

**Authors:** Amirreza Yousefzadeh, Evangelos Stromatias, Miguel Soto, Teresa Serrano-Gotarredona, Bernabé Linares-Barranco

**Affiliations:** Instituto de Microelectrónica de Sevilla (IMSE-CNM), CSIC and Universidad de Sevilla, Sevilla, Spain

**Keywords:** spiking neural networks, spike timing dependent plasticity, stochastic learning, feature extraction, neuromorphic systems

## Abstract

In computational neuroscience, synaptic plasticity learning rules are typically studied using the full 64-bit floating point precision computers provide. However, for dedicated hardware implementations, the precision used not only penalizes directly the required memory resources, but also the computing, communication, and energy resources. When it comes to hardware engineering, a key question is always to find the minimum number of necessary bits to keep the neurocomputational system working satisfactorily. Here we present some techniques and results obtained when limiting synaptic weights to 1-bit precision, applied to a Spike-Timing-Dependent-Plasticity (STDP) learning rule in Spiking Neural Networks (SNN). We first illustrate the 1-bit synapses STDP operation by replicating a classical biological experiment on visual orientation tuning, using a simple four neuron setup. After this, we apply 1-bit STDP learning to the hidden feature extraction layer of a 2-layer system, where for the second (and output) layer we use already reported SNN classifiers. The systems are tested on two spiking datasets: a Dynamic Vision Sensor (DVS) recorded poker card symbols dataset and a Poisson-distributed spike representation MNIST dataset version. Tests are performed using the in-house MegaSim event-driven behavioral simulator and by implementing the systems on FPGA (Field Programmable Gate Array) hardware.

## 1. Introduction

One goal of neuromorphic engineering is to map efficiently neurocomputational algorithms onto compact, low power, and fast hardware, while preserving satisfactorily the functionalities of the theoretical algorithms. The main first question (digital) hardware neuromorphic engineers ask themselves is about the minimum required bits to represent parameters and states. Theoretical neurocomputists, which use as tools conventional computers, use typically the full 64-bit floating point precision available to develop and study their algorithms. However, using 64-bit floating point precision for neuromorphic hardware, where typically massive parallelism is physically implemented, imposes a severe resources penalty not only for memory usage, but also for all computing and communication resources. Because of this, for example, the SpiNNaker spiking neuromorphic platform, although it uses 32-bit precision hardware, it restricts itself to integer arithmetic (instead of floating point), thus introducing some hardware simplifications (Furber et al., 2014). In another example, the TrueNorth platform (Cassidy et al., 2013) is built upon multiple 256 × 256 1-bit synaptic weight crossbars, although it includes extra circuitry to allow assigning up to four possible 8-bit values to the synapses (with some restrictions). In the world of non-spiking Deep Neural Networks (DNN), where there is now a strong quest for providing dedicated efficient hardware (Chen et al., 2016; Sim et al., 2016; Bong et al., 2017; Whatmough et al., 2017; Biswas and Chandrakasan, 2018; Gonugondla et al., 2018; Khwa et al., 2018), some theorists are studying ways to reduce bit precision of the weights down to 1-bit (Courbariaux et al., 2015; Rastegari et al., 2016) to help simplifying hardware. Here we focus on spiking neural network (SNN) hardware capable of on-line unsupervised learning through Spike-Time-Dependent-Plasticity (STDP). In a previous work (Yousefzadeh et al., 2017) we implemented deterministic STDP hardware with 9-bit resolution for the weights. Our goal here is to obtain a working learning (stochastic) STDP layer by restricting the weights to 1-bit precision (“0” or “1”) for both learning and inference phases. For this, we consider a feed-forward neural system made of two layers. The first layer, restricted to 1-bit weights, uses stochastic STDP unsupervised learning for learning representative features. The second layer uses some trainable SNN classifier with supervised learning for pattern classification (Stromatias et al., 2017; Yousefzadeh et al., 2017). Interestingly, the conventional full-precision additive STDP learning rule will result in a bimodal weight distribution after learning (Barbour et al., 2007; Galluppi et al., 2015). This means that, even in the case of graded synaptic weights, the final weight values tend to saturate to the minimum (disconnected) or maximum (fully connected) value.

STDP was originally proposed by Gerstner et al. (1993), evolving later on to successfully learn hidden spiking patterns (Masquelier et al., 2008), to perform competitive spike pattern learning (Masquelier et al., 2009), to achieve reward modulated (pseudo-supervised) learning (Mozafari et al., 2017), or to be successfully applied to deep spiking neural networks (Kheradpisheh et al., 2018). Surprisingly, experimental evidence of STDP in biological synapses was reported by neuroscientists shortly after proposing the computational algorithms for the first time (Markram et al., 1997; Bi and Poo, 1998, 2001; Zhang et al., 1998; Feldman, 2000; Mu and Poo, 2006; Cassenaer and Laurent, 2006; Jacob et al., 2007). However, STDP works typically by performing very small weight changes, which implies high resolution for the weights, consequently imposing high hardware resources demands for memory, computing, and communication circuits. Most of the neuromorphic hardware designs use on-chip memory to reduce power consumption (Cassidy et al., 2013; Davies et al., 2018)^1^ and therefore memory is a limiting factor for the number of neurons and synaptic connections. By using 1-bit synaptic weights, not only the memory, but all the processing elements inside the chip will be much simpler. Reducing weight resolution from 9-bit to 1-bit, should in principle allow for about one order of magnitude reduction in memory, computing, communication resources and power consumption. When restricting to 1-bit weights, one option is to consider some type of stochastic weight update. This is, instead of applying a given weight change from the STDP rule, one changes the weight from either “0” to “1” or from “1” to “0” with a probability given by the STDP rule. This idea has already been used before. Suri et al. (2013) applied it to cluster vehicle trajectories recorded with spiking retinas (Dynamic Vision Sensors -DVS-) (Lichtsteiner et al., 2008; Posch et al., 2011; Serrano-Gotarredona and Linares-Barranco, 2013; Son et al., 2017; Guo et al., 2017) into highway lane segments. Seo and Seok (2015) applied it to simple classification problems, but found out that they could not learn to separate more than five patterns. Here we want to learn to classify either DVS recorded poker card symbols (Soto, 2017) or a spiking representation of the MNIST dataset (LeCun et al., 1998). At the beginning, we were not able to obtain any reasonable learning by simply applying an STDP binary update with stochasticity. It was not until we started applying some additional “tricks” that we started to observe the formation of characteristic features together with overall reasonable accuracy results. These “tricks” were weight normalization, individual neuron threshold adjustment, or using separate thresholds for learning and inference.

Since there is no other work in the literature reporting classification accuracy results for 1-bit weights STDP feature extraction (FE) layers, we compare with purely random 1-bit weights FE layers. It is well-known that it is possible to build excellent performance pattern learning and classification systems by using a sufficiently large hidden FE layer with random weights, followed by a trainable high performance classification layer (Huang et al., 2006). Based on this, we compare our STDP systems with a “parallel” one using the same classifier output layer and a hidden layer with the same number of neurons but with 1-bit random weights. As shown later in the section 3, there is always a consistent improvement when using STDP with respect to using pure random weights, although this improvement reduces as the number of hidden layer neurons increases.

Throughout the paper we will use always a very simple neuron model, namely, the linear-leak (or piece-wise linear-leak) integrate-and-fire (LIF) model, together with instantaneous synapses (Camuñas-Mesa et al., 2010, 2011, 2012; Pérez-Carrasco et al., 2013; Serrano-Gotarredona et al., 2015), restricted to positive neural state values.

The paper is structured as follows. In the section 2 we will first include a quick review on different STDP rules, signaling the difference between more conventional time-based STDP rules vs. less conventional order-based STDP rules, which we will use here. Then we will briefly explain how we “engineer” STDP and, in particular, stochastic STDP with 1-bit weights by adding some “tricks.” Then we present quickly two previously reported spiking classifiers we have used, followed by a quick description of the software and hardware experimentation platforms used. In the section 3 we provide software and hardware results for three experiments. A first experiment is a very simple 4-neuron system that replicates a biological experiment of visual orientation tuning (Bienenstock et al., 1982; Moore and Freeman, 2012; Jeyabalaratnam et al., 2013). It is a very simple starting point used as reference in other computational studies (Galluppi et al., 2015). The second experiment learns to classify poker card symbols (Soto, 2017) recorded with a spiking retina Dynamic Vision Sensor (DVS) (Serrano-Gotarredona and Linares-Barranco, 2013). The third experiment learns to classify the MNIST dataset. More specifically, a spiking version of it, obtained by generating Poisson distributed spike trains from the original pixel gray levels. This is a common technique for converting static images to synthetic spike-trains and has been used in a number of previous studies (O'Connor et al., 2013; Querlioz et al., 2013; Diehl et al., 2015; Diehl and Cook, 2015; Galluppi et al., 2015; Stromatias et al., 2017). Finally, the paper finishes by presenting some Discussions and Conclusions.

## 2. Materials and methods

### 2.1. Review of some STDP rules

**Figure 1:**
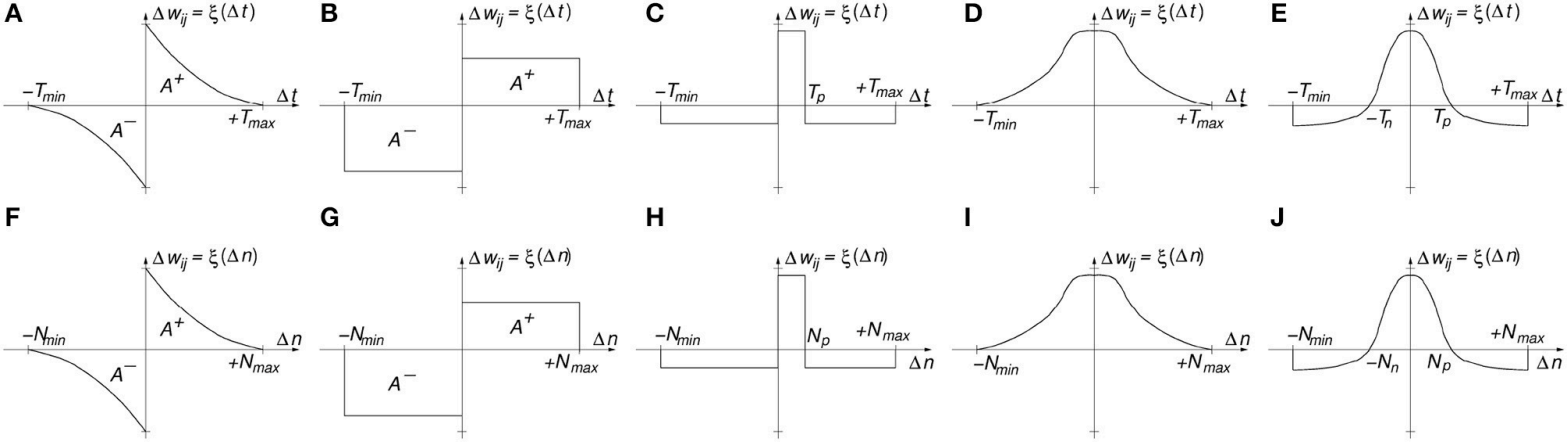
**(A)** Classic time-based STDP **(B)** Rectangular time-based STDP, **(C)** Narrow Potentiation time-based STDP, **(D)** Symmetric Hebbian Potentiating only time-based STDP, **(E)** Symmetric Hebbian with Narrow Potentiation time-based STDP, **(F)** Classic order-based STDP **(G)** Rectangular order-based STDP, **(H)** Narrow Potentiation order-based STDP, **(I)** Symmetric Hebbian Potentiating only order-based STDP, **(J)** Symmetric Hebbian with Narrow Potentiation order-based STDP.

Figure 1A shows a typical STDP update function ξ(Δ*t*), where Δ*t* is the time difference between a post-synaptic spike at time *t*_*post*_ and a pre-synaptic spike at time *t*_*pre*_. In biology, the time window [−*T*_*min*_, *T*_*max*_] is typically in the range of about 100 ms, although in artificial systems one can adjust this time window to the dynamics of the data spikes. Figures 1B–E illustrate other STDP learning functions ξ() that have been used in the computational neuroscience literature and/or observed in biology. For example, Figure 1B is a simplification of Figure 1A that provides a relatively similar outcome as long as the ratio of the areas under the positive *A*^+^ and negative *A*^−^ branches is preserved. Figure 1C shows an STDP learning function ξ() where potentiation is applied only for a narrow positive time window 0 < Δ*t*<*T*_*p*_, otherwise there will be depression. An interesting, powerful and widely used extension of this learning function is when +*T*_*max*_ → +∞ and −*T*_*min*_ → −∞ (Bichler et al., 2012; Suri et al., 2013; Querlioz et al., 2013). This is, whenever there is a post-synaptic spike, all synapses connecting to this destination neuron will be depressed by a fixed amount, except those who have transmitted a pre-synaptic spike during a prior time window *T*_*p*_ which will undergo a potentiation^2^. Let us call this type of STDP “undiscriminating depressing STDP.” This implementation requires less resources, as discussed later. Figure 1D is another interesting STDP version, where synapses are potentiated whenever pre- and post-synaptic spikes are more or less coincident in time, irrespective of which happens first. This “symmetric hebbian” STDP learning has also been found in some biological synapses (Roberts and Bell, 2002). Figure 1E is a type of symmetric “undiscriminating depressing” version of Figure 1D, also found in biology (Dan and Poo, 1998).

The STDP functions ξ(Δ*t*) in Figures 1A–F are all time-based (function of Δ*t*). In practical implementations (like digital HW or SW algorithms), one keeps a list of the pre- and post-synaptic spikes with their respective timestamps for later computation of the STDP function for each synaptic connection and corresponding update. Some neurocomputational researchers have proposed and successfully implemented time-abstracted versions of the STDP function ξ(), where the time variable has been removed and substituted by the order of the occurrence of the spikes (Thorpe and Gautrais, 1998; Masquelier and Thorpe, 2007; Bichler et al., 2012; Roclin et al., 2013). This way, each spike is not associated to a timestamp, but to an integer number *n* indicating its rank or order of occurrence. In this abstracted representation the time difference between consecutive spikes is irrelevant, and it only matters which spikes appear after or before each other. This is called “order-based” STDP and the learning function is now ξ(Δ*n*). In order-based STDP implementations one simply needs to keep track of an ordered list of pre- and post-synaptic spikes, without any timestamp. One can keep one single ordered list of the full system, separate ordered lists for sub-populations of the system, or even separate ordered lists for each synapse. When keeping track of the ordered lists, one should set a maximum number *N* of events to keep in the list, so that STDP learning is only driven by reasonably recent events. Figures 1F–J show the equivalent order-based STDP learning function versions of those shown in Figures 1A–E. One interesting feature of order-based STDP is that learning self-adapts to the dynamics of the neural activity. This is radically different to what happens in biology, where STDP is always time-based and consequently tuned to a specific range of time constants and dynamics. In order-based STDP the learning will occur in the same way, independently of the speed and dynamics with which events occur. If all spike timestamps are multiplied by the same constant, their ordering will be the same and consequently learning remains unchanged. Therefore, order-based STDP self-adapts to the speed and dynamics of the events.

### 2.2. Engineering STDP

**Figure 2:**
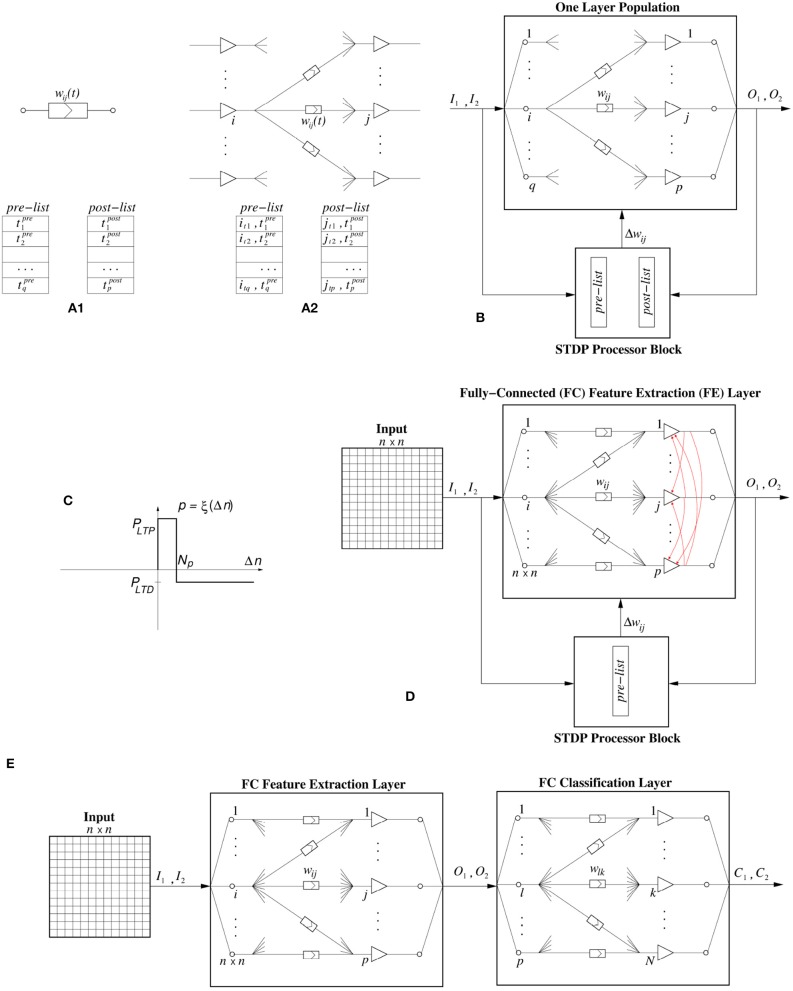
**(A)** STDP computation. **(A1)** By keeping a pre- and a post-synaptic event list per synapse, **(A2)** by keeping a pre- and a post-synaptic event list per neuron population with its input synapses. **(B)** STDP computation block assigned to a neuron population with its input synapses. **(C)** Stochastic STDP learning function. **(D)** The neural network topology used in this work for the fully-connected FE (feature extraction) hidden layer, where *p* is its size. The red arrows represent the lateral inhibition that is implemented internally. **(E)** Event-Driven System Topology used in this paper when the experiments require a FE layer and a Classifier layer.

In software as well as in hardware, STDP can be computed in many different ways. Here we follow an event-driven approach for both the software as well as the digital hardware implementation. This way, computations are not performed time-step by time-step, but only when an event is generated and transmitted. One way of computing STDP in an event-driven simulation is by keeping a bounded list of pre- and post-synaptic events. This is, for example, illustrated in Figure 2A1 for an individual synapse. Every time a pre-synaptic event is received by a synapse, its time of occurrence is stored in a pre-list or pre-buffer. If *t* is the present time, events are kept in the pre-synaptic list as long as $t-t_{n}^{pre}\leq T_{max}$. Similarly, whenever the post-synaptic neuron generates a spike, its time of occurrence is stored in a post-list or post-buffer, where events are kept as long as $t-t_{m}^{post}\leq T_{min}$. For every new pre-synaptic event at time *t*, it is stored in the pre-list, the post-list is scanned, and Δ*w*_*ij*_ is computed by using the corresponding $\xi \left(t_{m}^{post}-t\right)$ function. Similarly, for every new post-synaptic event at time *t*, it is stored in the post-list, the pre-list is scanned and Δ*w*_*ij*_ is computed using $\xi \left(t-t_{n}^{pre}\right)$. Depending on the type of STDP rule, one can choose to update a synapse for all the events in the list, or only for the most recent one(s).

However, keeping a separate pre- and post-list for each individual synapse is costly for both software and hardware implementations. Consequently, normally one keeps one pre- and one post-list for a full population, as illustrated in Figure 2A2, including the indexes *i* and *j* of pre- and post-synaptic neurons.

For the cases of order-based STDP rules, the pre- and post-list will not contain any time information, but just the indexes of the pre- and post-synaptic neurons in the proper order. The number of events kept in each list is a fixed integer number (which we will call “*buffer size*”).

Note that if one sets *T*_*min*_ = 0 or *N*_*min*_ = 0, only the pre-list is required. Similarly, if *T*_*max*_ = 0 or *N*_*max*_ = 0, only the post-list is required.

In the implementations (for both software and hardware) reported in this paper, we will keep a separate “STDP processor” connected to a neuron population containing its input synapses, as illustrated in Figure 2B. In this approach, the STDP processor receives a replica of, both, all pre-synaptic events *I*_*k*_ and all post-synaptic events *O*_*l*_. For each new event it checks the opposite list of stored events, and sends a kind of Δ*w*_*ij*_ command/event to the neuron population to update each of the *w*_*ij*_ weights that requires STDP update.

### 2.3. Engineering stochastic STDP with 1-bit weights

When restricting to 1-bit weights, it is no longer possible to consider small values for Δ*w*_*ij*_. Weights change directly from “0” to “1,” or vice versa. In this case, one can consider a stochastic version of STDP by simply substituting the learning functions in Figure 1 to provide a given signed probability^3^ for potentiation or depression. The weights are directly set to “1” (ON state) or “0” (OFF state) depending on the resulting probability and sign of function ξ(). For example, if ξ(Δ*t*) or ξ(Δ*n*) is equal to a positive probability *p* (with 0 ≤ *p* ≤ 1), then after generating a random number *x* (with 0 ≤ *x* ≤ 1), if *x* ≤ *p* the weight will be set to “1,” otherwise it will remain unchanged. In the case of negative probability *p* = ξ() values (with 0≥*p*≥−1), after generating the random number *x* (with 0 ≤ *x* ≤ 1), if *x* ≤ |*p*| the weight will be set to “0,” otherwise it will remain unchanged.

The proposed STDP implementation with stochastic updates is of the type “undiscriminating depressing” order-based STDP. Therefore, only the pre-list is kept and whenever a neuron generates a post-synaptic event, the synapses connecting to this neuron in the pre-list are potentiated while all remaining synapses connecting to this neuron are depressed. The STDP learning function is shown in Figure 2C, and is a particular case of Figure 1C for *N*_*min*_ = 0 and *N*_*max*_ = ∞, but with the vertical axis indicating either long term potentiation probability *P*_*LTP*_ or long term depression probability *P*_*LTD*_. Parameter *N*_*p*_ is the “buffer size” of the pre-list of the population, while Δ*n* is the event index in the pre-list of the population.

Additionally, we incorporate a number of additional “tricks” which help to obtain representative features^4^, while allowing the use of larger probabilities. This way, the learning is fast but stable. These “tricks” are the following:

1. **Individually incrementing neuron thresholds** *x*_*th*_*j*__. During learning, every time a neuron *j* produces an output event, its threshold *x*_*th*_*j*__ is incremented. This is because its receptive field weights will become more selective to a given feature, or equivalently, it has specialized to recognize a given feature, and consequently should give more chances to other neurons to pick up other features. To do so, its threshold is increased so that other still unspecialized (or less specialized) neurons have better chances to fire. This implements a form of *homeostasis* (Querlioz et al., 2013; Diehl and Cook, 2015). Typically, we increment a threshold by “1,” but it could be incremented by any predetermined integer number.
2. **Weight Normalization**. The sum of the weights $W_{sum}=\underset{i}{\sum }w_{ij}$ connecting to a neuron *j*, is maintained constant^5^. This can be enforced deterministically after each random update, or stochastically by readjusting the probability depending on the instantaneous value of *W*_*sum*_. If deterministic, the sum of weights will be kept constant at any time, but if stochastic, the sum will randomly wander around the target value. Without this “trick” some neurons become dead (null synapses) or over-activated (having too many active synapses), resulting in unstable learning. Different neuron populations can have different *W*_*sum*_.
3. **Neuron threshold** *x*_*th*_*j*__ **saturation**. The threshold *x*_*th*_*j*__ of each neuron in a plastic population is not allowed to exceed a predefined maximum threshold *x*_*th*_*max*__. This feature was introduced because we noticed that populations with fewer number of neurons for a given task produced very large final thresholds and ended up generating no events for an input sample during the testing phase.
4. **Separate neuron thresholds for STDP process and for inference process**. The idea is to keep for each neuron two different neuron states with their corresponding thresholds, one used for STDP weights updates and the other used for inference. Each incoming event updates both states. For inference the thresholds can be kept constant for simplicity, or they can also be adaptive as mentioned above. For STDP, the threshold is increased every time it is reached. Reaching the STDP threshold triggers weight update. By decoupling STDP and inference, neural activity can be kept high (which may improve learning at the next layer), while slowing down STDP learning as the neuron becomes more selective. Note that for STDP and inference processes to be decoupled, they should not have all characteristics equal (threshold increment, initial conditions). This “trick” is only implemented in the hardware implementations. The reason is that in the hardware versions, training is fully on-line. This is, all layers are trained simultaneously. Consequently, by separating inference and STDP thresholds, firing activity is kept high all the time, helping to accelerate learning in later layers. For the software versions, since learning is performed off-line, layers are trained sequentially.
5. **Lateral Inhibition**. When a population receives an input event, several neurons can reach their threshold and fire. However, we only allow one to fire. The rest of neurons in the population will be reset. This way we are implementing a form of internal lateral inhibition which resembles a winner-takes-all (WTA) (Maass, 2000) layer. This lateral antagonism forces competition between the neurons. It can be also more efficient for hardware designs, compared to the alternative method of using recurrent inhibitory soft connections.
6. **List Flushing**. After one output event is produced by a given neuron, the present list of stored pre-synaptic events of the population can be fully emptied. This way, the most recent history that triggered this output event is the only one used to contribute to learning for this output event. It will not contribute to learning induced by other output events. Although this “trick” had no impact on the learning of the systems tested (accuracy results are practically identical whether “list flushing” was active or not^6^), for the hardware implementations it introduced relevant simplifications, as explained later in the hardware description of the STDP unit.

In the software experiments, training was done off-line, layer by layer, thus decoupling the learning processes between layers. Therefore, as soon as the STDP learning process was finished in the FE layer, the weights were fixed to their final values and each neuron threshold (*x*_*th*_*j*__) was set to its last final threshold. Also, the lateral inhibition between neurons of the same population was disabled. After this, the classifier layer was trained independently. However, if an application requires a hardware implementation with learning, it is because on-line learning is required. Otherwise, one would implement a pure inferring hardware which is simpler and consequently more efficient (Chen et al., 2016; Sim et al., 2016; Bong et al., 2017; Whatmough et al., 2017; Biswas and Chandrakasan, 2018; Khwa et al., 2018). Therefore, for our hardware tests we always implemented simultaneous on-line learning for all layers.

### 2.4. Network architecture

The neural network topology used throughout this work can be seen in Figure 2D. It is a fully connected topology, where the first layer is the input layer, whose size *n*×*n* depends on the size of the particular data set. The second layer, which is a fully connected (FC) Feature Extraction (FE) layer, consists of a population of spiking neurons. Attached to the plastic FE neural layer is the stochastic binary weighted STDP processor block which receives two inputs, one from the presynaptic input layer and one from the post-synaptic FE layer. The STDP block sends weight update events Δ*w*_*ij*_ with the indexes of the synapses that need to be potentiated, as well as, a special Δ*w* event that will trigger the undiscriminating depression.

### 2.5. Event-based classifier

Some of the experimental results shown later are classification problems which, after the 1-bit weights stochastic STDP feature extraction layer, require an additional event-driven classifier layer, as shown in Figure 2E. We have used two different event-driven (SNN) classifiers. One is a very simple low performance one which uses a kind of supervised STDP training rule (Yousefzadeh et al., 2017). In this case, it is possible to train simultaneously the two layers, by making the classifier layer learn slower. We have followed this approach for our hardware experiments. The second classifier is a high performance one that needs to be trained off-line in the frame-domain (Stromatias et al., 2017). In this case, one needs to train first the STDP layer and once it has finished learning, the classifier layer is trained off-line. We have followed this approach in our software experiments. More details of each classifier are as follows:

1. **Simple Supervised STDP-based SNN Classifier**. This classifier is trained following a conventional non-binary non-stochastic STDP learning rule (Yousefzadeh et al., 2017), in which the post-synaptic spikes are replaced by a teacher signal indicating which is the correct category. The sum of weights of synapses connecting to an output neuron is kept constant (weights are normalized after each weight update).
2. **High-Performance SNN Classifier**. For training this classifier, the method is to present the spike-trains of each training and testing sample and store the normalized spike-counts (histograms) of the FE layer output for each input sample. This way we create a new data set of frames that have somehow captured the dynamic activity (spike count per symbol) of the FE layer. The advantage of creating this new data set of frames is that we can now train a fully connected Softmax classifier in the frame domain using Stochastic Gradient Descent (SGD) (Bottou, 2010). When the frame-based classifier is trained, it can be converted to a population of LIF neurons by scaling the classifier's weights by a constant *k* and setting the threshold of the LIF neurons to *k*. It has been shown there is a small loss when converting from the frame-based to the SNN classifier that is in the order of 0.03% for synthetic data and 0.68% for real DVS sensory data (Stromatias et al., 2017).

It is worth mentioning that a very efficient classifier layer can compensate for a poor feature extraction hidden layer. As a matter of fact, the extreme learning learning machine (Huang et al., 2006) is one such an example. It is capable of performing excellent classification by using a hidden layer with random weights but with a large number of neurons, followed by a high performance classifier. Therefore, the benefit of having a better feature extraction hidden layer is more evident with a lower performance classifier or with small number of neurons in the hidden layer.

### 2.6. Experimentation platforms

Experiments were performed using two different platforms. One is an Event-Driven software simulation tool called MegaSim, and the other is a hardware implementation using a commercial FPGA.

#### 2.6.1. Megasim software simulator

MegaSim (*Modular Event-Driven Growing Asynchronous Simulator*^7^) is a tool designed for behavioral event-driven simulations of multi-module hardware systems, with strong emphasis on the low-level hardware details of the modules such as processing delays, handshaking, communication delays, parameter variations, etc. The user can add new modules by adding descriptions in C-language, allowing therefore for arbitrary model complexities. This simulator has already been used in prior work (Stromatias et al., 2017), and more details about its internal operations are available in the Appendix.

#### 2.6.2. Hardware implementation

**Figure 3:**
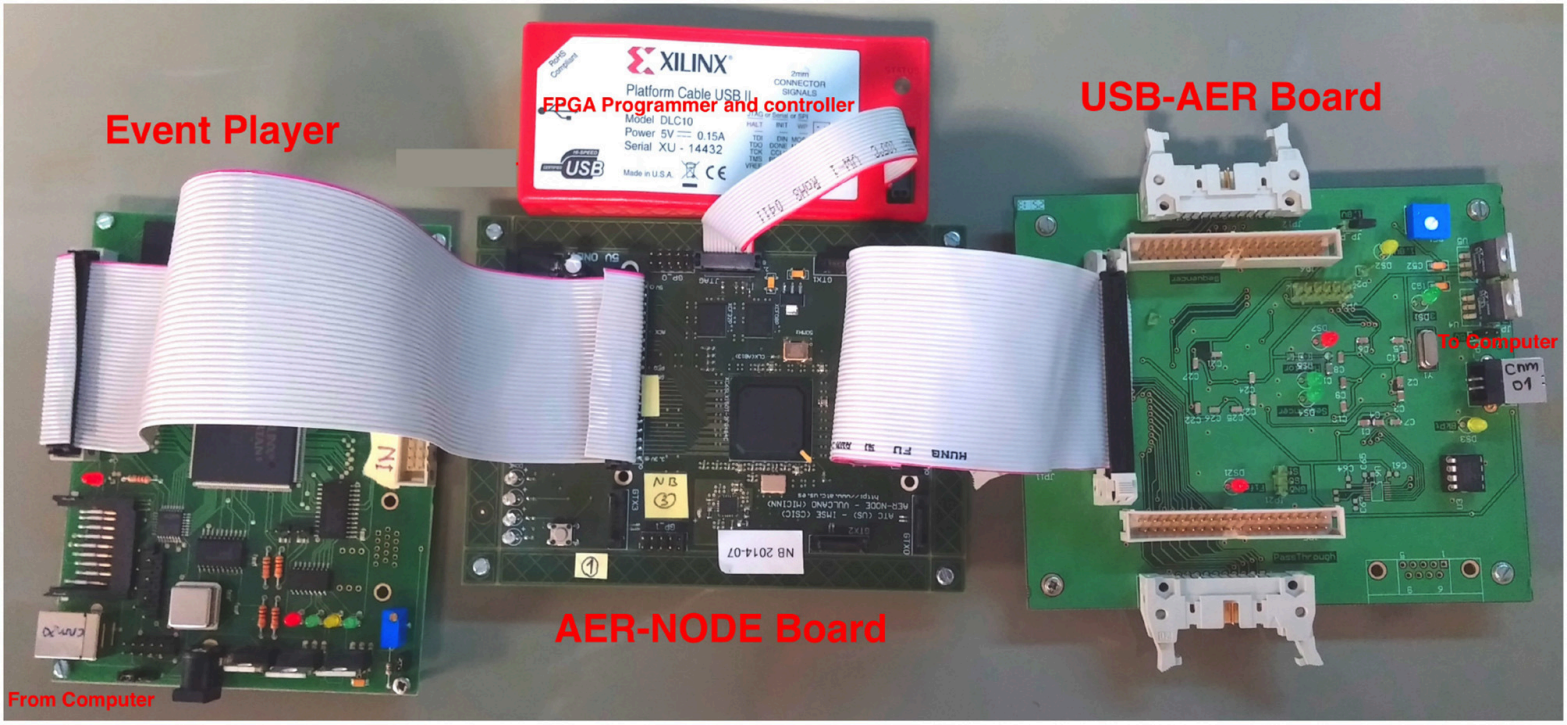
Hardware setup for demonstration of on-line real-time Stochastic Binary-Weights STDP learning. This setup contains an Event-Player (USBAER board) (Serrano-Gotarredona et al., 2009) to play back recorded AER events with precise timing, an AER-NODE board (Iakymchuk et al., 2014) which contains the Spartan-6 (XC6SLX150T-3) FPGA and a computer interfacing board (USBAERmini2) (Serrano-Gotarredona et al., 2009) to send the output AER events along with their time-stamps to a computer through USB. The computer uses jAER (Delbruck, 2007) to visualize and/or record events in real time displaying them as sequences of frames on a monitor screen.

The hardware platform we are using here for Stochastic 1-bit weights STDP allows for on-line unsupervised learning of the FE layer, with simultaneous on-line supervised STDP-based classifier learning (Yousefzadeh et al., 2017). This digital hardware has been designed using standard Hardware Description Language (HDL) and is implemented on a Spartan-6 based FPGA (XC6SLX150T-3) custom platform. Figure 3 shows the hardware setup used^8^.

Two main blocks have been developed for the hardware implementation on digital FPGAs: a *neuron block* and an *STDP unit*. The *STDP unit* can be shared by several *neuron blocks*, as STDP processing has much lower occurrence. The inhibition mechanism prevents two or more neurons from simultaneously learning a pattern. Therefore, at the most one single neuron will be requesting usage of the STDP unit at a given time. Additionally, by separating firing and STDP thresholds, STDP thresholds are reached with lower frequency than average firing rate. Consequently, the STDP unit will be requested with much less frequency than the neuron updates. Therefore, one single STDP unit can be shared by several neurons. In our hardware implementation, several neurons with one *STDP unit* form a *neural core*. Next we describe each block.

##### 2.6.2.1. Neuron block

**Figure 4:**
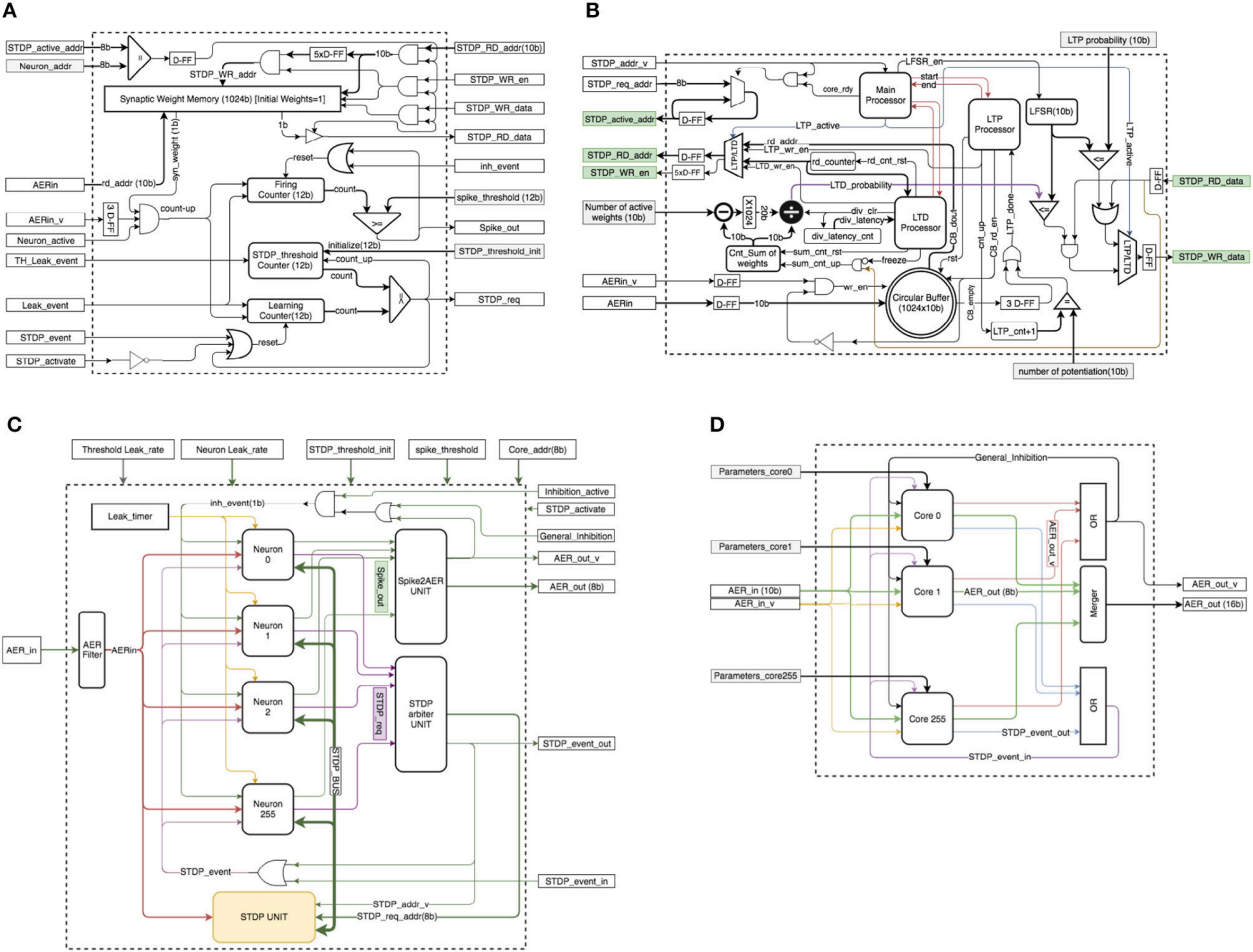
**(A)** Neuron block schematics. **(B)** STDP unit schematics. **(C)** Neural core schematics. **(D)** Multi-core configuration.

Figure 4A shows the structure of a neuron block. The implemented neuron block contains a 1024-bit synaptic RAM to keep the values of up to 1024 binary synapses, three counters (“Firing Counter,” “STDP_threshold Counter,” and “Learning Counter”), some D-type flip-flops (D-FF), three logic comparators, and a few digital gates. The 1024-bit synaptic memory has two independent ports, one is used inside the neuron block (*syn_weight*) to read synaptic weights and the other one is connected to an STDP bus (*STDP_RD_data*) for STDP type of operations. When a new spike comes in, signal port “*AERin_v*” will be activated. “*AERin*” contains the address of the incoming spike. The corresponding synaptic weight for the incoming spike will be read from the synaptic memory, which requires 3 clock cycles. If the synaptic weight is one, then the state of the neuron should be increased by one, by asserting “*count_up*.” For the neuron implementation we keep two separate states, each with its own counter, the “*Firing Counter*” for inference and the “*Learning Counter*” for learning. The “*STDP threshold Counter*” stores the threshold for the STDP events. After each learning/STDP process, the value of the STDP threshold is increased by one. The values of the “*Learning Counter*” and the “*Firing Counter*” are constantly compared with their corresponding threshold values to generate an STDP event (“*STDP_req*”) or an output event/spike (*Spike_out*), respectively. The neuron states are reset to zero if they exceed the threshold or an external reset event inhibits them because of lateral inhibition. When a neuron asserts the “*STDP_req*” signal, the external STDP unit will access the synaptic memory (through “*STDP_active_addr*”) to perform the learning process. Since the STDP unit is shared by multiple neurons, neurons are connected to a shared bus, which we call *STDP_BUS* (see Figure 4C), made of signals {*STDP_active_addr, STDP_RD_add, STDP_WR_en, STDP_WR_data and STDP_RD_data*}. *Neuron_addr* is unique for each neuron. When the STDP unit wants to access a neuron's weight, it will put the address of the neuron in the *STDP_active_addr* line. Each neuron can process one input spike per clock cycle, because the 3-cycle reading from synaptic memory is pipelined.

In a Spartan-6 FPGA (XC6SLX150T), each neuron occupies only 56 slices (out of 23 K) and it can be clocked at up to 300 MHz [therefore, each neuron can process up to 300 Meps (mega events per second)]^9^. This allows to implement more than 400 physical neurons and 400,000 synapses in this FPGA without using block-RAMs.

##### 2.6.2.2. STDP unit

The STDP unit is significantly more complex than the neuron block. Figure 4B shows a detailed block diagram for the STDP unit. This unit contains a circular buffer to memorize the addresses of up to the latest 1024 incoming spikes (which we called “pre-list” or “pre-Buffer” in section 2.2), three processors (Main Processor, LTP Processor, LTD Processor), a 10-bit random number generator (LFSR), four counters (*rd_counter, div_latency_cnt, Cnt_Sum of weights, LTP_cnt+1*), a subtracter logic block, a × 1024 multiplier block (implemented as a 10-bit shift), a divider block, three logic comparators, three multiplexers, a number of D-FF registers, and digital gates.

Parameter “*number of potentiation*” indicates how many events in the circular pre-Buffer will be actually used (“*number of potentiation*” ≤ 1024). This information is used for the LTP (long term potentiation) process. The main processor acts as a general manager. Activation of signal *STDP_addr_v* means that the STDP unit has received an STDP request. In this case, if the main processor is ready to process a new request, it puts the address of the selected neuron on the *STDP_active_addr* line. Then it asserts the start signal for the LTP processor. After finishing the LTP process, it will assert the start signal for the LTD (long term depression) processor. The first step of the STDP process is LTP. In this step, the LTP processor controls the *STDP BUS* of the selected neuron. It reads the addresses of the most recent spikes that are stored in the circular buffer and puts them on the *STDP_RD_addr* bus. Then, after reading the synaptic weight, the value needs to be potentiated with a pre-defined probability. In this case, a 16-bit LFSR generates a pseudo-random number. It compares 10 (out of 16) bits of this random number with the LTP probability value, and if the random number is smaller than the probability value, the synaptic weight will be potentiated to “1” if it was “0” before. This process repeats until the number of LTP actions becomes equal to parameter “*number of potentiation*.” Each potentiation can be done in one clock cycle but there is a 3 clock cycle latency for reading from the circular buffer and the same for reading from the synaptic memory of the neuron. One clock cycle latency is also added for the *STDP_WR_data* bus to make a proper pipeline. Therefore, in total, the LTP process requires 7+num_potentiation clock cycles, which in the worst case is 1024+7=1031 clock cycles. After the LTP process, the LTD process starts to depress some weights randomly and normalizes the number of active weights in a synaptic memory. The LTD processor first reads all the synaptic weights of the selected neuron to compute the sum of the weights and calculates the depression probability. This will take 1024+3 clock cycles. Then a subtracter logic block calculates the difference between the number of actual active weights and the number of expected active weights (Δ*W*). After this, a logic 10 bit shift multiplies Δ*W* by 2^10^ to scale it. Then a divider logic block calculates the normalized LTD probability with the following formula

(1) $$\begin{array}{r} \text{LTD Probability}=\frac{1024\times \Delta W}{W_{sum}} \end{array}$$

where *W*_*sum*_ is the number of actual active weights. This process requires 25 clock cycles because the divider logic is very small and it is a slow serial divider. Since only one division is needed for the whole STDP process, it is efficient to use a slow and small divider. After calculating the LTD probability, the LTD processor reads again all the synaptic weights, one by one, and depresses them using this probability. This step requires 1024+7 clock cycles. Therefore, in total, the LTD process requires 1024+3+25+1024+7 = 2083 clock cycles.

At the end of the STDP process, all the pointers in the circular buffer will be reset. This implements pre-list flushing. In HW, lateral inhibition is very strong by directly resetting all neurons within the same population sharing the same STDP UNIT. Consequently, it does not make sense to keep the old events in the circular buffer for future STDP. This simplifies dramatically the HW. Otherwise, if lateral inhibition would have been soft, we may think of not flushing the pre-list. But this would require to add the following complication: since the STDP process is much slower than the neuron updates, while an STDP update is running, new incoming events should not be included in the circular buffer before the running STDP process is finished, requiring the introduction of an intermediate buffer whose content would be transferred to the circular buffer once the STDP update process is concluded.

The proposed design implemented in Spartan-6 (XC6SLX150T-3) consumes 120 slices (out of 23k), and it can operate at a clock frequency of up to 200 MHz^10^. The exact time of the STDP process depends on one parameter (*number of potentiation*), which in the worst case needs 2083+1031 = 3114 clock cycles, or 15μ*s*.

As a practical consideration, a VLSI design needs a hierarchical approach to be extensible and reusable. Here we implemented a *Neural-Core* as a single processing core that contains an arbitrary number of 256 neurons and one STDP unit. Figure 4C shows the block diagram of this *Neural-Core*. Each core contains two arbiters. One arbiter (“*STDP arbiter UNIT*”) is in charge of handling STDP requests from the neurons, while the other arbiter (“*Spike2AER UNIT*”) is in charge of handling the output spikes produced by the neurons. Since the number of neurons per core can be large, the use of the shared bus (“*STDP_BUS*”) reduces long-distance wiring resources. There are only 2 signals (1-bit each) that need to be routed separately for each neuron: “*Spike_out*” and “*STDP_req*.” An AER filter is implemented at the “*AERin*” input to filter out all input spikes whose destination is not the present Neural Core. The Neural Core can have any arbitrary number of neuron units, as long as their output activity does not saturate the shared “*STDP UNIT*.”

##### 2.6.2.3. Multi-core configuration

Figure 4D illustrates an arrangement of a multi-core configuration for the case of a one-layer fully connected neural network with an arbitrary number of 256 cores (65 k neurons). This can be one layer of a deep neural network. In this case each core can have its own parameters.

**Figure 5:**
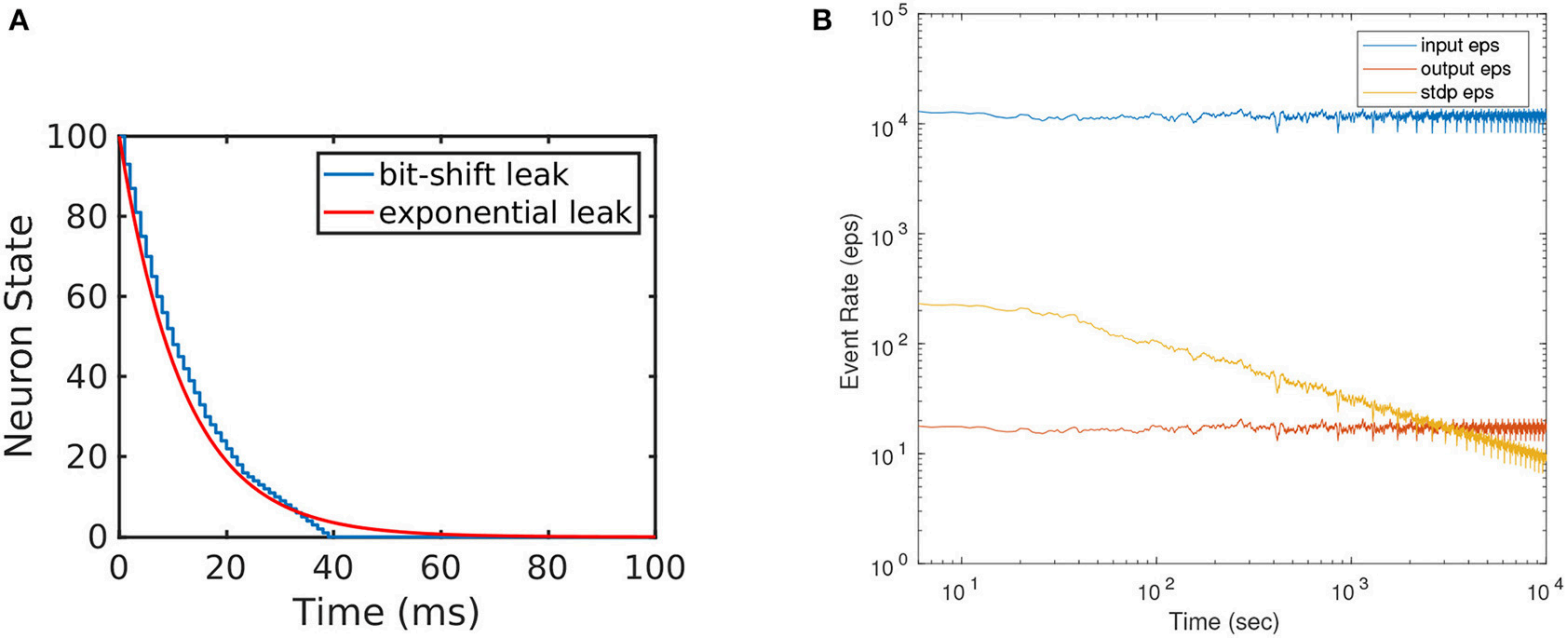
**(A)** Illustration of the implemented efficient leakage mechanism by using bitwise shift. This approximates exponential leakage in LIF neurons. **(B)** Event rate of input, output, and STDP output during STDP learning. Horizontal axis (time) is shown in log scale to visualize the exponential decay in STDP output events. One training set trial is about 400 s.

The efficiency of the presented hardware implementation comes from the simplicity of the neuron model, of the learning rule, and from using 1-bit synaptic weights. We approximate the exponential leak by a simplified piece-wise-linear leak, by using a bit-wise shift mechanism for leakage^11^(Yousefzadeh et al., 2017). Figure 5A shows the exact exponential leak (with 12 ms time constant), together with the approximated piece-wise-linear one. Initially, a large number is subtracted every time (fast linear leak), but this number is divided by a power of 2 at some point, reducing the slope of the linear leak. Additionally, the simplicity of the order-based STDP learning rule avoids keeping track of time, saving on-chip memory. For a comparison, Rice et al. (2009) implemented 25 physical Izhikevich neurons (without any learning scheme) in a Virtex4 FPGA, consuming 79% of total resources (more than 2 k slices per each physical neuron) which is around 40 times more than the resources used for the simple neurons used in this work. Cassidy et al. (2007) presented an FPGA design with 32 LIF neurons and 128 8-bit synaptic connections per neuron which are equipped with conventional time-based STDP learning and operates at 50 MHz clock frequency. Their design consumed 7454 slices of Xilinx Spartan-3 FPGA and 20 KB of block RAMs while our proposed design with 256 neurons (including STDP unit and clocking at 100MHz) consumed 6210 slices and no block RAMs^12^. This means that by using 1-bit weights, we were able to integrate more than 8 times smaller neurons with 8 times more connections per neuron, while having almost identical resource consumption.

## 3. Results

This section describes three learning experiments performed on three different data sets, using either the MegaSim software platform, the FPGA hardware platform, or both. The first one is an experiment on the unsupervised development of simple orientation selectivity fields replicating biological experiments (Bienenstock et al., 1982). These biological experiments demonstrate the development of stimulus orientation selectivity in the primary visual cortex. To replicate this biological experiment no classifier layer was used, and results were obtained for both the MegaSim software platform and the hardware FPGA platform. The second experiment is a poker card symbol classification experiment using real DVS recordings. We used the so called “Slow Poker DVS” data set (Soto, 2017), which consists of relatively long recordings of hand held printed poker symbols, that allowed for a reasonable large training set. This experiment required a classification layer, and we used both, the high performance and the simple STDP-based ones. Results were obtained for the MegaSim and the FPGA platforms. The third experiment is an MNIST handwritten digits (LeCun et al., 1998) recognition experiment. For this experiment we required the high performance classifier, as the simple classifier provided poor performance for such a complex data set. Results were only obtained for the MegaSim platform, because the high performance classifier cannot be trained on-line by the hardware. In the following we show details of the different experiments and corresponding results.

### 3.1. Development of orientation selectivity

**Figure 6:**
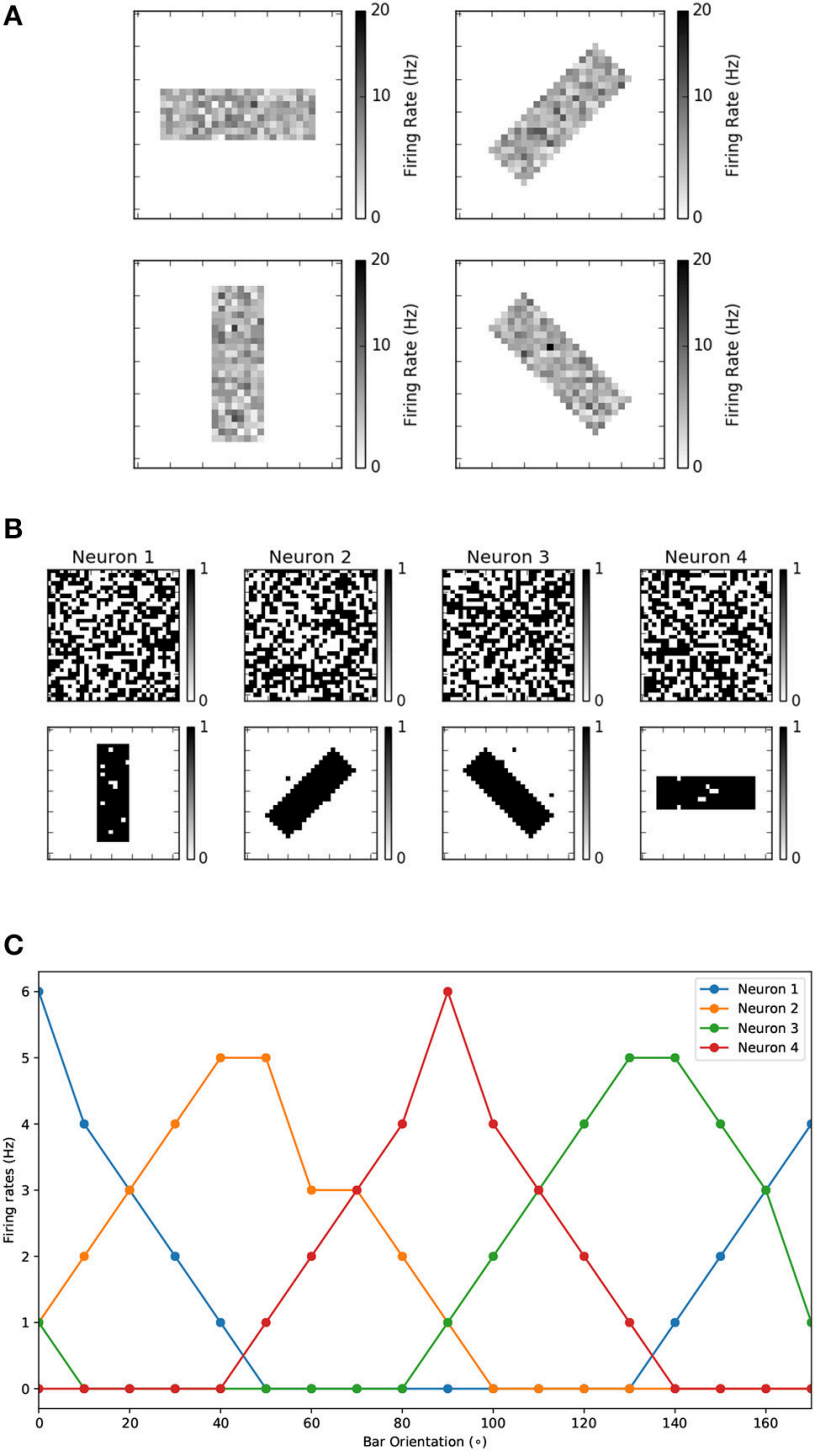
**(A)** The input pattern used for the orientation selectivity experiment. During the experiments the bar is presented in random order. **(B)** The initial and final 1-bit weights for the development of orientation selectivity experiment. **(C)** Orientation tuning curves obtained by rotating a horizontal bar counter clockwise with a step size of 10°C.

This experiment is based on the work presented in Bienenstock et al. (1982) and was used to experimentally demonstrate the development of stimulus selectivity in the primary sensory cortex. The topology of this neural network was shown in Figure 2D. It consists of two layers, one input layer of size 32 × 32, which is fully connected to a FE layer of 4 neurons. The synthetic stimulus used for the training is a bar presented in 4 different orientations, rotated by 45^*o*^. The bar is 8 pixels thick, and 24 pixels in length, while each pixel has a random intensity value between 0.8 and 1.0. The pixels are then converted to Poisson spike-trains with a rate proportional to the intensity of each individual pixel. This is a common technique for converting static images to synthetic spike-trains (O'Connor et al., 2013; Querlioz et al., 2013; Diehl et al., 2015; Diehl and Cook, 2015; Galluppi et al., 2015; Stromatias et al., 2017). The four different orientations used for the training can be seen in Figure 6A. During training, each bar is presented in one of the four random orientations, with an inter-symbol time equal to *T*_*leak*_, thus allowing all neurons to leak to their resting state.

The parameters of the fully connected neural population are summarized in Table 1 top, while the STDP parameters can be seen in Table 1 bottom. Figure 6B top shows the initial random 1-bit weights before learning, and Figure 6B bottom shows the final 1-bit weights after 400 training epochs. It can be seen that by the end of the training each neuron has developed a receptive field specific to a particular orientation. The purpose of the post-learning test is to demonstrate that each neuron has learned to respond maximally to a preferred orientation. For this, we disabled the (a) plasticity, (b) dynamic thresholds, and (c) internal (inhibition) reset mechanism. Neuron thresholds are kept at their final training values (which is *x*_*t*_*h*__*max*__).

**Table 1:** Neural and STDP parameters for the development of orientation selectivity experiment.

**Parameters**	**Values**	**Description**
**Neural parameters**		
*population*_*size*	4	Population size
*pre*_*size*	1024	presynaptic population size
*x*_*th*_	10	Initial Neuron Threshold
*x*_*th*_*max*__	100	Maximum allowed dynamic threshold
**STDP parameters**		
*STDPbuffersize*	250	Total size of the event buffer
*P*_*LTP*_	80%	Probability for LTP
*W*_*sum*_	180	Sum of active synapses per neuron

This experiment has been performed both on the MegaSim and on the FPGA platforms, presenting identical results. Figure 6B shows the firing frequencies of the four neurons as a function of input stimulus bar orientation, in steps of 10°. As the bar rotates, the neuron tuned to the closest orientation provides higher firing frequency, as happens in biology (Bienenstock et al., 1982) and other similar experiments (Moore and Freeman, 2012; Jeyabalaratnam et al., 2013; Galluppi et al., 2015). For the rest of the paper, in the remaining experiments, we will consider the spike count of the output neurons, for a given input stimulus, as the quantity determining the readout of the neurons.

In the FPGA, we implemented four physical neurons and one STDP UNIT. The complete circuit power consumption was 142 mW when operated at 100 MHz clock frequency. This design occupied only 1% of the FPGA slices. A video demonstration of the FPGA version of this experiment can be seen in (Yousefzadeh, 2017b).

### 3.2. Recognition of DVS-recorded poker card symbols

A very high speed DVS recorded poker symbol data set was released in the past (Serrano-Gotarredona and Linares-Barranco, 2015). However, this data set contained too few training samples for proper STDP learning and classifier training. To perform more robust poker symbol recognition training, a new data set was recorded called “*Slow-Poker-DVS*,” in which paper printed poker symbols were hand held in front of a DVS and moved slowly to induce motion events (Soto, 2017). This data set consists of four 3 min recordings, each recording for a different poker symbol. The recordings use the full 128 × 128 DVS resolution. For each recording, we extracted slices with a fixed number of events per slice, rather than with a fixed time window. More specifically, we used slices of sizes 10, 30, and 50 kilo events per slice (kesl). Therefore, we had three different data sets for training and testing, depending on the chosen kesl. Each slice is not like a static frame, but rather like a moving video, and a few neurons may fire and learn during each slice. For finding the optimal learning hyper parameters such as *W*_*sum*_, *x*_*t*_*h*__*max*__, STDP Buffer Size, and *P*_*LTP*_, we used a validation set to avoid over-fitting the testing set (Nowotny, 2014).

The number of samples of the training and testing set varies depending on the number of kesl. For 10 kesl, we had 779 samples in the training set (of which 138 samples are used for the validation set) and 192 samples in the test set. For 30 kesl we had 259 samples in the training set (of which 45 are used as the validation set) and 64 samples in the test set. Finally, for 50 kesl we had 155 samples in the training set (of which 26 samples are used for the validation set) and 37 samples in the test set. For each experiment the hyper parameters that generated the highest score on the validation set were used to train on the full training set and report the final score as CA on the testing set.

#### 3.2.1. Software results

For each data set we trained the SNN with different number of neurons in the feature extraction layer, the layer that will learn with 1-bit weights. The number of neurons we tried for this layer were {200, 400, and 800}. The experiments were executed by applying a *P*_*LTP*_ of 20 and 80%, a number of active synapses per receptive field *W*_*sum*_ of {16, 32, 128}, and *x*_*t*_*h*__*max*__ of {40, 60}. The STDP buffer size was kept fixed at 250 events to reduce simulation time.

**Figure 7:**
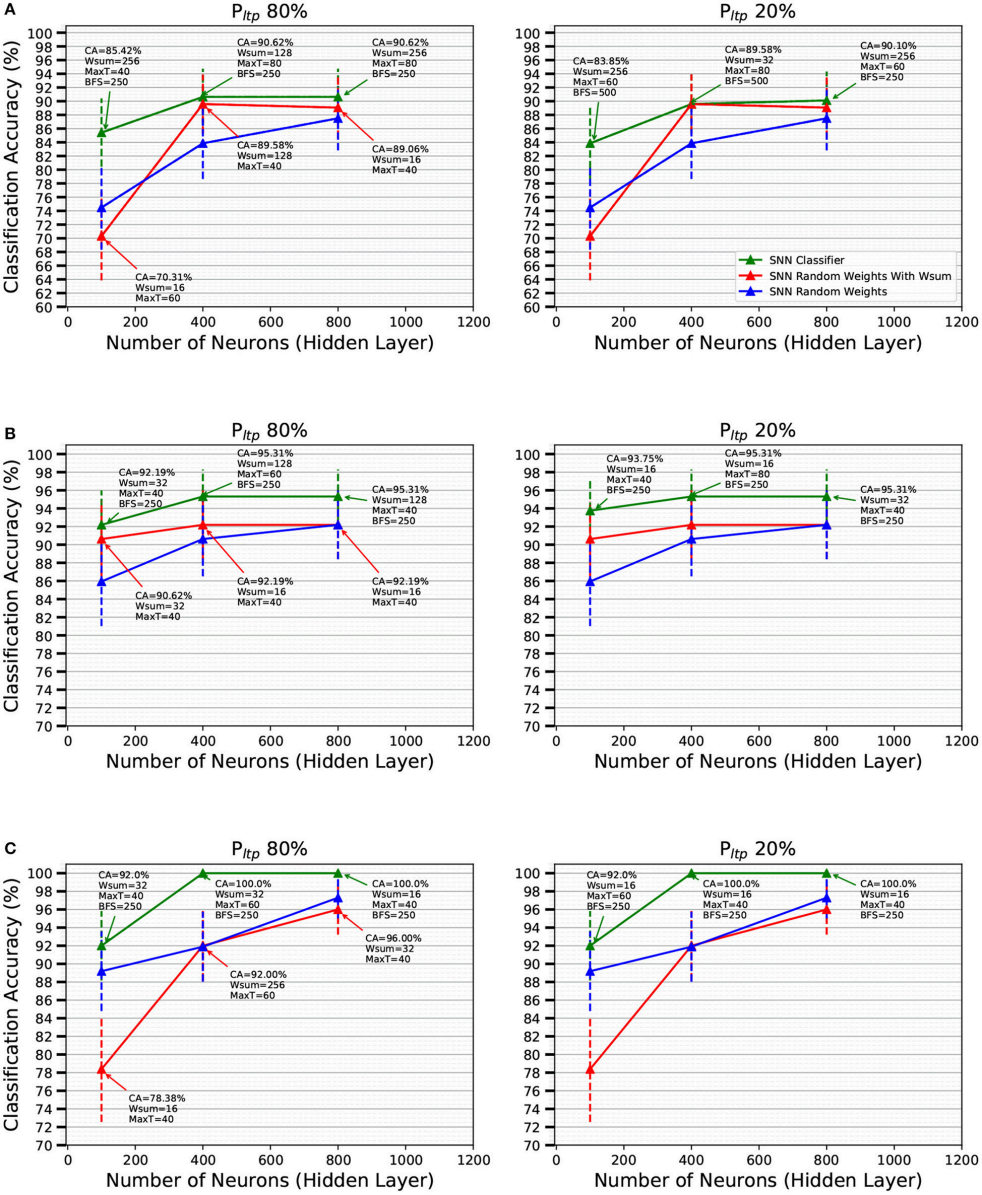
Classification Accuracy (CA) of the SNNs on the Slow-Poker-DVS data set as a function of the number of neurons in the FE layer for a *P*_*LTP*_ of 80 and 20%, shown as the green solid line, for **(A)** 10 kesl, **(B)** 30 kesl, and **(C)** 50 kesl. The text at each data point presents the hyper parameters that yielded the best CA on the validation set. The blue solid lines show the CA for random fixed 1-bit weights with no STDP learning in the FE layer, while the red lines show the CA for random fixed 1-bit weights with fixed *W*_*sum*_ applied to restrict the number of active synapses per neuron. *P*_*LTP*_ is not applicable for the blue and red solid lines; they are duplicated in each pair of subplots for viewing convenience. The error bars denote the confidence and are calculated for a confidence level of 0.99 and assuming that the test samples are statistically independent (Nowotny, 2014).

In order to find the optimal hyper parameters, we used the validation set of each data set. For each data set, each training set is presented once with stochastic STDP learning activated. Then, the hyper parameters that resulted in the highest classification accuracy (CA) on the validation set were chosen to train with the full training set, and after this the classifier (output layer) was trained. The final score is reported using the testing set of each data set. Table 2 gathers the hyper parameters that were investigated for the Slow-Poker-DVS experiments, when using the MegaSim platform with the high performance event-driven classifier (Stromatias et al., 2017). The corresponding results are summarized in Figure 7. The green line represents CA on the corresponding testing set as a function of the number of neurons in the 1-bit weights feature extraction layer. Figures 7A–C correspond to data sets created using 10, 30, and 50 kesl, respectively. It can be seen that there is no significant difference between processing most of the incoming STDP updates (*P*_*LTP*_ = 80%) or just a portion of them (*P*_*LTP*_ = 20%). Although CA improves with more neurons in the feature extraction layer, the number of kesl has a more significant effect on the CA. Along with the CA, Figure 7 presents the confidence interval (CI) for each result with a vertical bar. The CI is calculated for a confidence level of 0.99 and assuming that the test samples are statistically independent (Nowotny, 2014). The confidence intervals show the expected CA depending on the test set size. The confidence intervals of the mean test error are computed using the following expression (Nowotny, 2014)

(2) $$\hat{\mu }\pm z^{*}\left(\sqrt{\frac{\hat{\mu }(1-\hat{\mu )}}{N_{T}}}\right)$$

where $\hat{\mu }$ is the estimated test error, *N*_*T*_ is the test set size, and z^*^ depends on the level of confidence. For 0.99 confidence level, z^*^ is 2.578. Note that the resulting range of the CI is quite wide because the data set used is not very large. Furthermore, with the increase of kesl, the number of samples of the data set and the test set becomes smaller and the CI increases.

**Table 2:** STDP hyper parameters investigated for the MNIST classification task.

**Parameter**	**Values**	**Description**
*STDP buffer size*	[250, 500]	STDP event buffer size
*W*_*sum*_	[16, 32, 128, 256]	Number of active synapses per neuron
*P*_*LTP*_	[80%, 20%]	Potentiation probability
*x*_*t*_*h*__*max*__	[40, 60, 80]	Maximum allowed threshold during STDP learning

The “*Slow-Poker-DVS*” data set was used before with the same high performance classifier, but using a feature extraction layer of 18 pre-programmed non-plastic oriented Gabor filters (Stromatias et al., 2017), obtaining a CA of 99.7%. Slices were extracted using 100 ms time windows, generating a total of 6751 samples for the full data set. Here we decided to be independent of the time variable and create slices using number of events. However, the large number of events required for a good CA, results in a reduced number of samples in the data set, which increases the CI for each result. Using fixed time slices we obtained 99.7% CA (Stromatias et al., 2017). On the other hand, here the data set got reduced drastically, but we achieved even better CA, despite using 1-bit weights, a smaller network size, and training the feature extraction layer in an unsupervised manner.

In order to “measure” the effectiveness of the STDP-trained FE layer, we compare with the case of using pure random 1-bit weights in the FE layer. It is well-known that a sufficiently large FE layer with random weights followed by a high-performance classifier yields highly competitive accuracies (Huang et al., 2006). Here we want to see if stochastic STDP learning with 1-bit weights improves with respect to pure random 1-bit weights, using the same FE layer size. The results are shown in Figure 7 with blue and red traces. Blue traces correspond to generating random 1-bit weights without any restriction. Red traces correspond to the same, but with the restriction of keeping the sum of 1-bit weights (connecting to one destination neuron) equal to *W*_*sum*_. We can see there is a systematic benefit when using stochastic STDP with respect to random weights. The benefit tends to be more for smaller number of neurons in the FE layer.

#### 3.2.2. Hardware results

**Figure 8:**
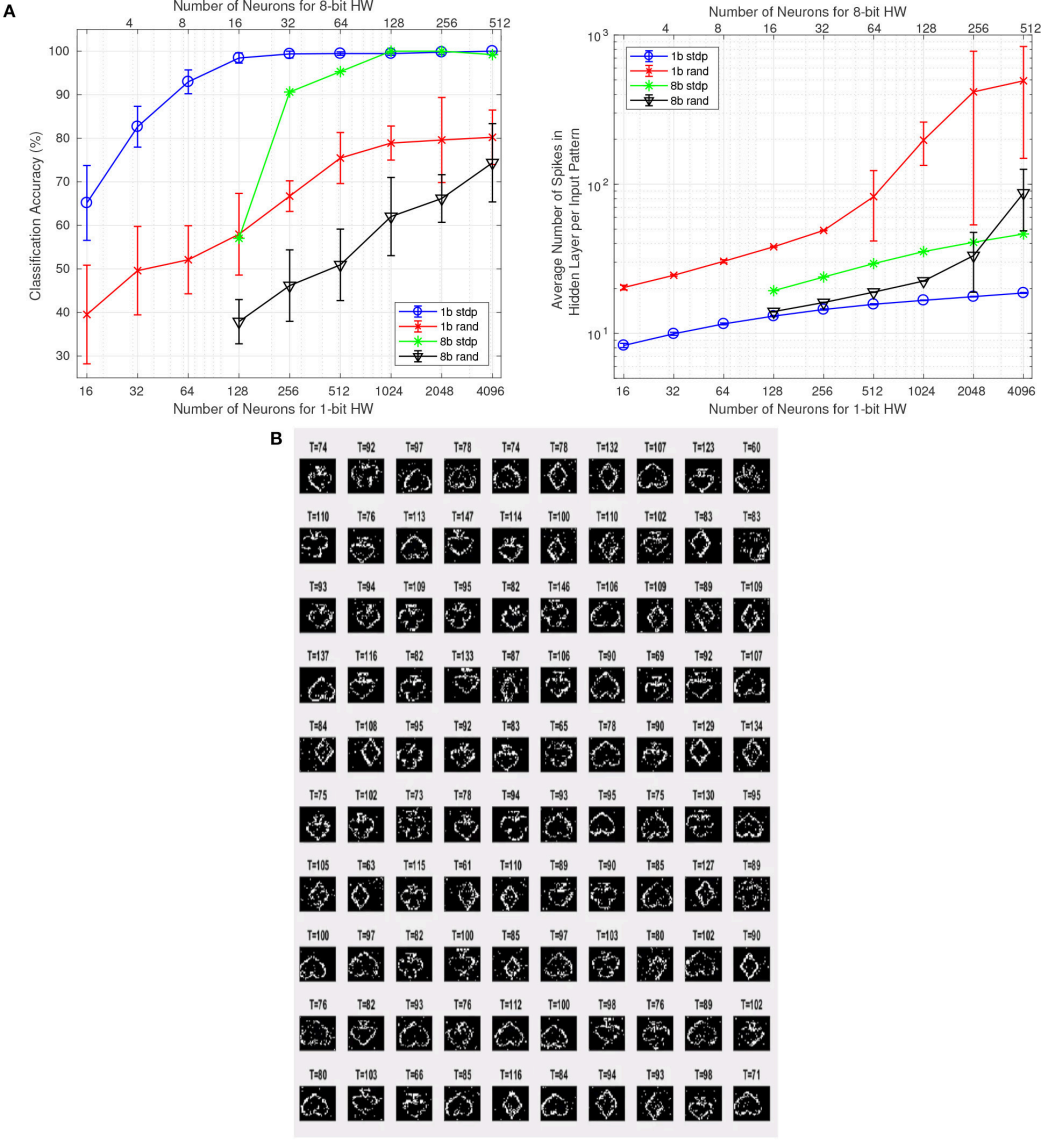
**(A)** CA (left) and output neuron spikes (right) for STDP trained FE weights and random FE weights. The figure compares 1-bit hardware (bottom axes) with 8-bit hardware (top axes). Bottom and top axes are aligned for similar hardware resources consumption. Blue circles: 1-bit HW with STDP. Red exes: 1-bit HW with fixed random weights. Green asteriscs: 8-bit HW with STDP. Black triangles: 8-bit HW with fixed random weights. **(B)** Stochastic-STDP learned weights on the FPGA hardware platform for buffer size 90 and *P*_*LTP*_ = 30%. The number on top of each receptive field indicates the final neuron threshold *x*_*th*_ after learning has concluded.

In addition to the MegaSim based experiments with the high performance classifier, we also performed some tests using the digital hardware FPGA platform with the simplified event-driven classifier. The FPGA platform results shown in Figure 8A (trace “1b stdp”) correspond to the 10kesl data set, with input events sub-sampled to 32 × 32 pixel resolution. From each original recording, we used the first 1.6 million events, which resulted in 160 samples per symbol. From these, 80% randomly picked samples were used for the training set and the remaining 20% for the test set. We set up a network with a 32 × 32 input layer which connects to a feature extraction (FE) layer with *N* neurons in a fully connected manner. The 32 × 32 × *N* 1-bit synaptic weights were trained on-line using the proposed stochastic STDP 1-bit rule with *W*_*sum*_ = 100. The *N* neurons are connected to a simple STDP-type classifier (as presented before in section 2.5) with 4 output neurons, and trained accordingly (Yousefzadeh et al., 2017). This classifier is implemented in hardware and is trained on-line along with the stochastic STDP neurons. We performed tests using different numbers of neurons *N* for the FE layer. Also, for comparison purposes, we repeated the same process but without STDP FE learning. Instead, the receptive fields of the *N* FE neurons were randomly set to 1-bit values, while keeping *W*_*sum*_ = 100. Figure 8A (trace “1b rand”) shows the results when setting the event buffer size to 90 and the probability of potentiation to *P*_*LTP*_ = 30%. The bottom axes in Figure 8A indicate *N*, the number of neurons in the FE hidden layer. Figure 8A (left) indicates the CA obtained after stochastic STDP learning and classifier training, and Figure 8A (right) indicates the average number of FE hidden layer spikes per pattern presentation for the full test set. We can see that when using the proposed stochastic STDP FE layer learning, the classifier can learn with up to 100% accuracy with *N* = 256, while generating much less events. However, when the FE weights are set randomly, the classifier is not able to achieve such high accuracy. Figure 8B shows the stochastic-STDP learned FE 1-bit weights for^13^ *N* = 100. For these results, we kept for all *N* the same STDP buffer size (90), *W*_*sum*_ (100), and *P*_*LTP*_ (30%). However, the inference threshold was automatically adjusted to have an acceptable activity for classification. In general, a higher activity should lead to improved accuracy as there is more information. However, we can see that for the STDP FE layer, higher accuracy is achieved with lower neural activity (and consequently lower power consumption), which means that extracted features are more efficient and representative. For this experiment, with 256 physical neurons and one STDP UNIT, the FPGA consumed 333 mW when operating at 100 MHz clock frequency. Such design occupied 27% of our FPGA slices.

#### 3.2.3. Trading off bit-resolution for number of neurons while maintaining similar hardware resources

An interesting question one may ask is what if, for the same hardware resources, we increase bit-precision while reducing number of neurons and synapses. Will we gain in CA or power consumption? Changing bit-precision affects all circuitry: synapses, neurons, as well as all computing and communication circuitry. Although a full detailed study is out of the scope of the present paper, we can quickly analyze the case for an 8-bit system, using both STDP or fixed random 8-bit weights. For example, as reported by Cassidy (Cassidy et al., 2007), they implemented an STDP 8-bit system using very similar hardware resources than our 1-bit STDP implementation (see **Table 5**). The number of synapses and neurons they were able to fit was exactly 1/8 the ones we have used. Therefore, as an approximate rule of thumb, we can suggest that increasing/decreasing bit resolution by a given number, decreases/increases both the number of synapses and neurons in the hidden layer by the same number. This observation is actually quite intuitive when implementing a hidden layer with *N*_*i*_ inputs, *N*_*h*_ hidden neurons, and *N*_*syn*_ = *N*_*i*_×*N*_*h*_ synapses. If *R*_*h*_ represents the hardware resources for the hidden layer neurons, *R*_*syn*_ the ones for the synapses, and *R*_*ov*_ the ones for the rest of overhead (shared) circuitry, then

(3) $$\begin{array}{rcl} R_{syn} & = & \alpha_{syn}\times N_{i}\times N_{h}\times n_{b} \end{array}$$

(4) $$\begin{array}{rcl} R_{h} & = & \alpha_{h}\times N_{h}\times n_{b} \end{array}$$

(5) $$\begin{array}{rcl} R_{ov} & = & \alpha_{ov}\times n_{b} \end{array}$$

where *n*_*b*_ is the number of bits, and α_*syn*_, α_*h*_, and α_*ov*_ are some proportionality constants. Assuming *R*_*ov*_ < < *R*_*syn*_+*R*_*h*_, then we can see that the product *N*_*h*_×*n*_*b*_ must be kept constant for equal hardware resources. Consequently, we may assume that if we change bit resolution from 1-bit to 8-bit, we must divide *N*_*h*_ by 8. In Figure 8A we show CA (left) and Average Number of Hidden Layer Spikes per Input Pattern (right) as a function of the number of neurons for different implementations. The horizontal axes have been separated into two: the bottom one for 1-bit hardware and the top one for 8-bit hardware. This way, hardware of similar resources is aligned vertically. For example, a 1-bit implementation with 256 hidden layer neurons is aligned vertically with an 8-bit implementation of 32 neurons, because both consume similar hardware resources. Vertical segments at each data point, represents the max/min spread when repeating the training over 10 full trials. For the case of 8-bit STDP training there are no vertical segments, because in this case STDP is deterministic: STDP applies small changes every time without generating random numbers in the process.

From Figure 8A we can make some interesting observations. (a) The 1-bit hardware with STDP provides, for given hardware resources, the highest CA and the minimum number of spikes in the hidden layer. Note that the number of spikes is directly proportional to power consumption in an event-driven system, because computation and information communication is performed at a per-event basis. Also note that computing events in 1-bit hardware consumes less energy than with 8-bit hardware (although, this is not reflected in Figure 8A). (b) CA seems to depend mainly on the number of neurons, while depending very little on the bit representation used. In Figure 8A left, when shifting the 8-bit HW curves to align exactly the number of neurons, the resulting curves almost overlap. (c) For the fixed random weights (both 1-bit and 8-bit) there is a sudden increase in number of events as hidden layer neurons increase, while for the STDP cases the tendency seems to be to settle smoothly. Please note that none of these observations is conclusive, and should not be extrapolated to more general cases before a more careful and systematic study is performed.

#### 3.2.4. Bandwidth limitations of STDP hardware during learning

From section 2.6.2 we can see that the hardware resource usage of the STDP unit within one 256-neuron core (see Figure 4C) is about 2-3 neurons, or equivalently, about 1% of the neural core. Therefore, from the hardware resources point of view, the difference between a 1-bit STDP hardware and a 1-bit fixed random weights hardware is negligible. On the other hand, the biggest advantage of the fixed random weights option is that it does not require training. From the bandwidth point of view, the STDP version has some bandwidth limitations during learning. For inference, both versions have identical bandwidth because the neuron hardware is the same. Neurons can process one event per clock cycle. Therefore, for the 100 MHz clock frequency implementation (see section 3.2.2) the maximum input event throughput is 100 Meps. Additionally, the maximum output event throughput cannot exceed 100Meps, for the same reason.

However, the STDP version needs to be trained and, during training, the STDP unit imposes some additional bandwidth limitation, which can be reached under some circumstances. In our example implementation, the STDP unit is shared by 256 neurons, and is triggered every time one of the 256 neurons generates an STDP output event. Each STDP output event also resets the STDP counters of the rest of neurons, thus helping to make the STDP output activity sparse. As mentioned in section 3.2.2, the STDP process needs “2090 + buffer size” clock cycles. In our example implementation “buffer size = 90,” thus requiring 2180 clock cycles per output event (21.8μ*s* at 100 MHz clock frequency). Therefore, the STDP unit would saturate at an STDP output event rate of 45.87 keps. Note that saturating the STDP unit is not a dramatic problem: while saturated, all STDP output events will be ignored and training would require more time. Figure 5B shows, as an illustration for the 256-neuron hidden FE layer case, the instantaneous event rates for the input flow (average around 12 keps), for the output flow (average around 18 eps), and for the output STDP events. We can see that STDP output event rate starts with a maximum of about 220 eps and decreases exponentially with time as learning progresses. Consequently, one could accelerate the input event rate by a factor 45.87*k*/220 = 208 before saturating the STDP unit during the initial learning phase, which would result in an input event rate of 2.5 Meps. Alternatively, if one wants to avoid STDP unit saturation, it is possible to add more STDP units and have them shared by less neurons.

### 3.3. Recognition on the MNIST data set

The MNIST data set (LeCun et al., 1998) consists of 70,000 samples of handwritten digits from 0 to 9, of which 60,000 are used for training and 10,000 for testing. Each sample is a 28 × 28 gray-scale image with maximum intensity value of 255. We converted the MNIST data set from static images to Poisson-distributed spike-trains with a rate proportional to the intensity of each pixel, while all firing rates are scaled in order to keep the total firing rate of the population constant (O'Connor et al., 2013; Stromatias et al., 2017, 2015).

MNIST dataset learning was only possible when using the high performance classifier for the output layer. Otherwise, accuracy results were very poor. Consequently, here we only focus on software platform results with the high-performance classifier. During STDP learning we presented one sample at a time, 1000 spikes per training sample, with an inter-symbol-time (IST) identical to the linear leakage of the spiking neurons. This way the leakage acts as a natural way to reset the internal states of the neurons before presenting a new training sample.

We first train the FE layer using the topology shown in Figure 2D. Stochastic STDP learning stops when all training samples are presented once. Training was repeated for different number of neurons in the FE layer. For the output layer we used the high performance event-driven classifier described in section 2.5. For finding the optimal learning hyper parameters such as *W*_*sum*_, *x*_*t*_*h*__*max*__, STDP Buffer Size, and *P*_*LTP*_, we used a validation set to avoid over-fitting the testing set (Nowotny, 2014). We split the original 60, 000 samples of the full training set into a training set of 50, 000 samples and used the remaining 10, 000 samples as the validation set. For each experiment the hyper parameters that generated the highest score on the validation set were used to train on the full 60, 000 samples training set and we report the final score as CA on the MNIST testing set.

**Figure 9:**
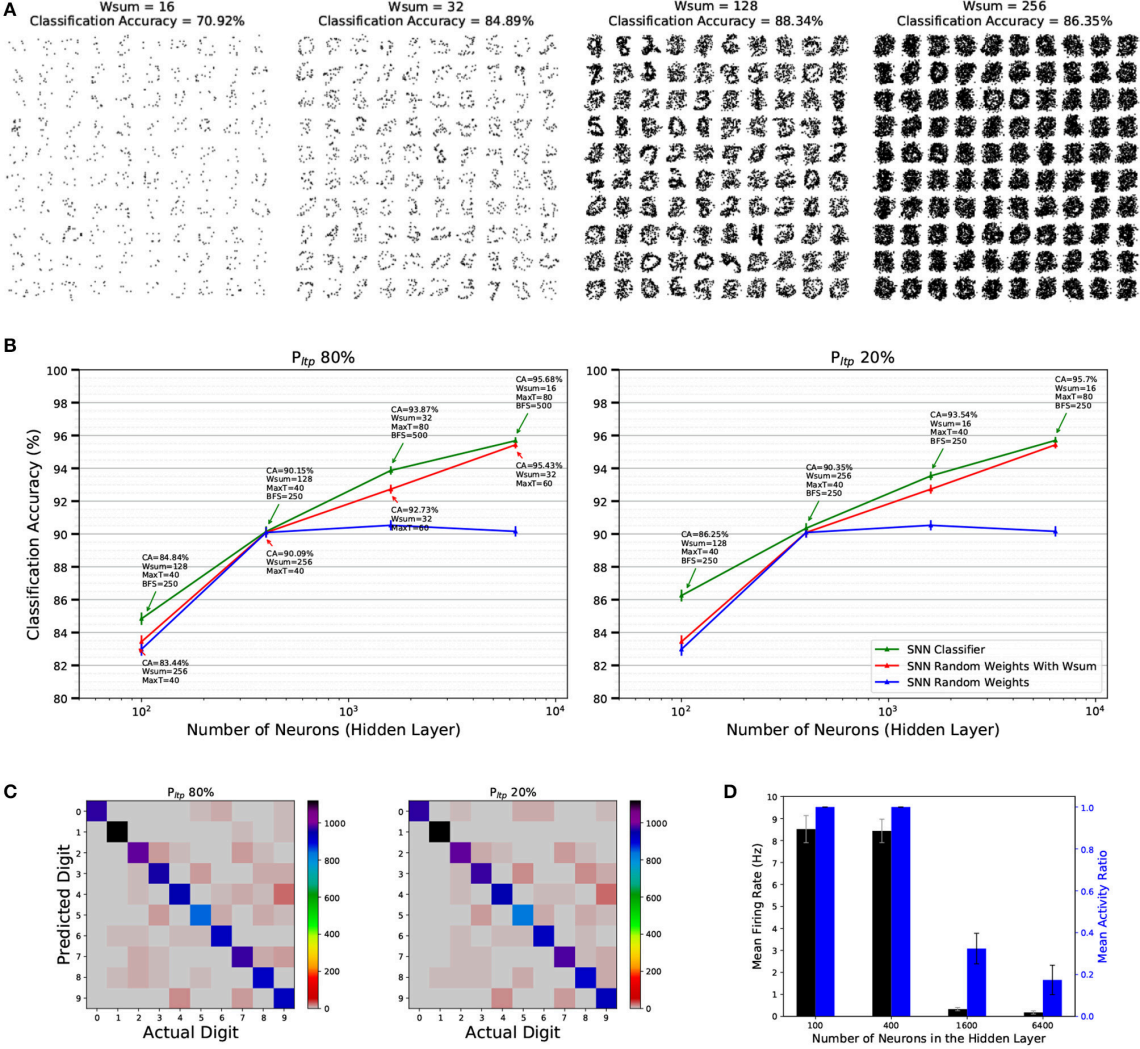
**(A)** Effect of *W*_*sum*_ Stochastic-STDP meta parameter on the 1-bit weights (receptive fields) and the classification accuracy (CA) of a population of 100 neurons trained on the MNIST data set. Weights are reshaped from 784 to 28 × 28. **(B)** Classification Accuracy (CA) of the SNNs on the MNIST testing set as a function of the number of neurons in the FE layer for a *P*_*LTP*_ of 80 and 20%, shown as the green solid line. The text at each data point presents the hyper parameters that yielded the best CA on the validation set. The blue solid line shows the CA for random fixed 1-bit weights with no STDP learning in the FE layer, while the red line shows the CA for random fixed 1-bit weights with fixed *W*_*sum*_ applied to restrict the number of active synapses per neuron. Error bars denote the confidence and are calculated for a confidence level of 0.99 and assuming that the test samples are statistically independent (Nowotny, 2014). **(C)** The confusion matrix on the 10, 000 MNIST testing set digits for a FE layer of 6400 spiking neurons when applied STDP learning with a P_*LTP*_ of 80 and 20% respectively. Values on the diagonal represent correct classifications. **(D)** Mean neuron firing rate and mean activity ratio of the FE layer for all 10, 000 samples of the validation set and for 4 different population sizes.

We trained a series of SNNs with varying number of neurons in the stochastic STDP FE layer. The number of neurons were {100, 400, 1600, 6400}, as in (Diehl and Cook, 2015) to allow for a direct comparison. Table 2 shows the STDP hyper parameters that were investigated. Figure 9A shows the effect that *W*_*sum*_ has on the learned weights of a population of 100 neurons. Results are summarized in Figure 9B. The green solid line in Figure 9B represents CA on the MNIST testing set as a function of the number of neurons in the FE layer, along with the hyper parameters that generated the highest CA on the validation set. For *P*_*LTP*_ = 80% the SNNs achieve the following classification accuracies for populations of 100, 400, 1600, and 6400 neurons in the FE layer: 84.84, 90.15, 93.87, and 95.68%, while for a *P*_*LTP*_ = 20% CA is 86.25, 90.35, 93.54, 95.7%. These results demonstrate that there is no significant difference between processing the majority of incoming STDP weight updates or just a fraction of them. Another visible trend from Figure 9B is that, as the number of neurons in the FE layer increases, configurations with smaller *W*_*sum*_ (less active synapses per neuron) yield higher classification accuracies, while for smaller population sizes larger *W*_*sum*_ result in higher CA. Figure 9B presents the CA along with the confidence intervals (CI).

Figure 9C shows the confusion matrix of the MNIST test set for the 6,400 neurons case for a *P*_*LTP*_ of 80 and 20%. Values on the diagonal represent correct classifications. While executing simulations with the validation set we discovered that the activity of the FE layer after stochastic STDP learning for the larger populations is very sparse and the average firing rate is very low. For example, the mean firing rate of the FE layer for 6400 neurons on the validation set is 0.26 Hz, while less than 20% of the neurons are active per sample on average. These findings are summarized in Figure 9(D).

As before, results were compared with pure random 1-bit weights, without normalization (blue traces in Figure 9B), and with normalization (red traces in Figure 9B). We can see again a systematic beneficial trend when using STDP with respect to using random weights, which is clearer now than for the Poker dataset case, since the confidence intervals are now much narrower. The benefit tends to be larger for smaller number of neurons in the FE layer, as in the previous case.

As a final experiment, we used the simple classifier for the output layer in order to investigate if the reason why the results produced with 1-bit weights STDP and random 1-bit weights with *W*_*sum*_ are similar is because of the high-performance classifier being too powerful or our proposed stochastic binary STDP not generating good features. For a FE layer of 1600 neurons and for features learned with stochastic 1-bit weights STDP the simple classifier achieved a CA of 75.6%, while for random 1-bit weights with the same *W*_*sum*_ = 16 the CA was 47.6%. These results verified our hypothesis that the reason why the results we achieved with the 1-bit weights STDP and random 1-bit weights with *W*_*sum*_ show similar trend is because our high-performance classifier is indeed too powerful and overcomes the poor features generated randomly. On the other hand, when using the simpler but lower-performance classifier, stochastic STDP provides better features, improving overall CA with respect to using random weights.

## 4. Discussion

There is a growing interest in exploiting SNNs for practical hardware applications, not only because they approximate better the inherent operations of the brain, but also because only meaningful information, represented by spikes/events, consume computing resources and energy. Originally, there was almost no work on training SNNs directly in the spiking domain, and many research efforts were inverted in efficiently mapping conventional (frame-driven) ANNs (Artificial Neural Networks), conveniently trained in the frame-domain, to their SNN counterpart (O'Connor et al., 2013; Pérez-Carrasco et al., 2013; Diehl et al., 2015). However, recently, there has been a growing success in training networks directly in the spiking domain, either by developing some type of backpropagation technique for the spiking domain (Lee et al., 2016; Neftci et al., 2017; Mostafa, 2018), by exploiting STDP at some level of the network (Querlioz et al., 2013; Diehl and Cook, 2015; Neftci et al., 2015, 2016; Kheradpisheh et al., 2016), or by some *ad-hoc* technique (Lagorce et al., 2016; Negri, 2018).

For the simple 4-class DVS-recorded poker card recognition datasets (Serrano-Gotarredona and Linares-Barranco, 2015; Soto, 2017), reported results are summarized in Table 3. There are two poker card dataset versions. The fast one (Serrano-Gotarredona and Linares-Barranco, 2015) of visual field resolution 32 × 32 pixels, where symbols cross the screen in about 20–30 ms. And the slow one (Soto, 2017) of visual field resolution 128 × 128 pixels, where symbols move slowly and recordings are cut into sequences of about between 0.75 (10 kesl) and 3.75 (50 kesl) seconds. The fast set has been used by several researchers. In the original paper using the fast dataset (Pérez-Carrasco et al., 2013), 91% of CA was achieved with around the first 500 events, which corresponds to about the first 1–3 ms of a recording. This was achieved by training a 3-layer ConvNet in the frame-domain using backpropagation and then mapping it to an SNN. Orchard (Orchard et al., 2015) used a 1-layer ConvNet with 18 Gabor filters with a heuristic classification method, achieving 97.5% CA after processing the full recordings, each with about 5 k events. The HOTS technique (Lagorce et al., 2016) based on computing with space-time surfaces, achieved 100% CA by processing also the full recordings. Recently, another mapping method from the frame-domain to SNN was reported (Kaiser et al., 2017) based on applying Contrastive Divergence (CD) on a hidden layer of a generative model with 10 convolutional feature maps, achieving 91% CA after processing the full recordings. Negri developed a method based on histrogramming, which is capable of recognizing extremely fast (0.6 ms or less than 50 events), with a reasonable accuracy of 96%. Stromatias et al. (Stromatias et al., 2017) used a 1-layer ConvNet with 18 Gabor filters followed by an SNN classifier. They achieved 99.77% CA on the fast set and 100% on the slow set. For all the above methods, synaptic weights used the full resolution of 64-bit floating point precision, except for (Stromatias et al., 2017) who used integer precision of 24-bit for the classifier layer and 32-bit for the convolution layer. In this work we used just 1-bit precision for the first layer, trained with STDP directly in the spiking domain, with the same 24-bit integer resolution classifier layer than (Stromatias et al., 2017), achieving 100% CA on the slow set. Unfortunately, we could not use the fast set because we needed more training samples for STDP.

**Table 3:** Comparison of reported results on DVS-recorded Poker datasets (“Fast” and “Slow”).

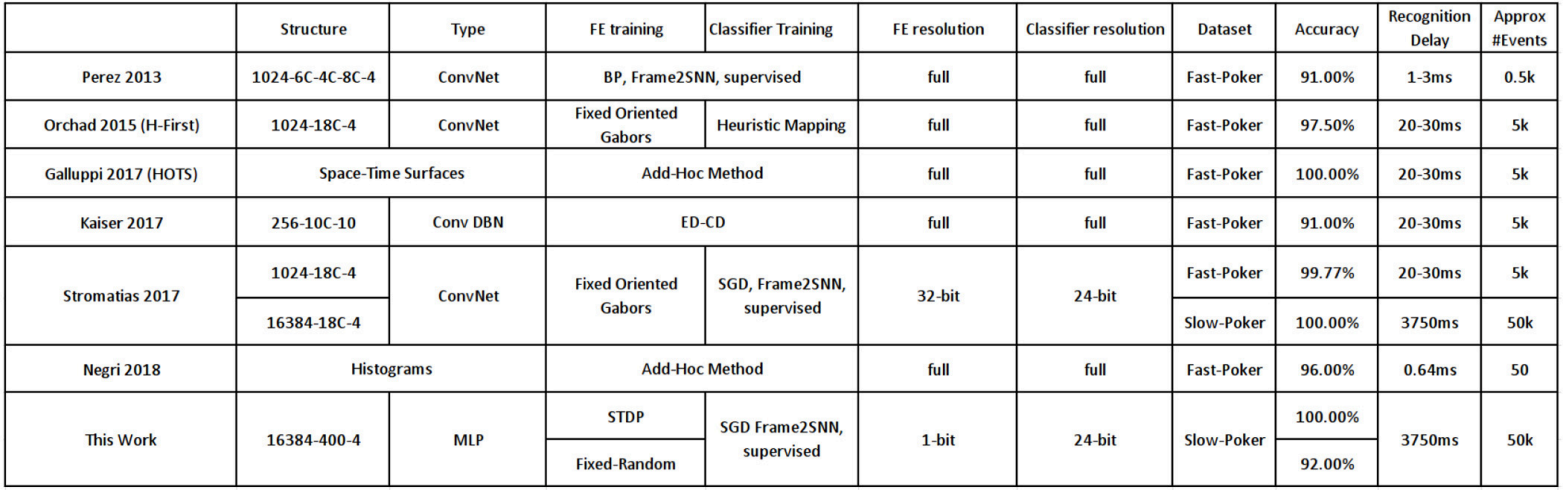

Table 4 shows SNN results reported for spiking versions of the MNIST dataset. As indicated in the last column, the original frame-based data set is converted artificially into spikes by either mapping a pixel value into an average frequency of a spike train with Poisson distribution, by mapping the pixel value to a delay (resulting in one spike per pixel), or by flashing the MNIST digit on a monitor while recording spikes with a DVS spiking retina sensor (O'Connor et al., 2013)^14^. Table 4 is divided in two parts by a double horizontal line. The top part uses synaptic weights with high resolution, while for the bottom part synapse weight resolution has been reduced to 8-bit or less. The entries shown in red correspond to networks that have used STDP for some of their weights training. The second last column indicates whether the models are generative or not^15^. Column “Structure” indicates the number of layers of each system and the number of neurons (or convolutional feature maps) per layer. Column “Type” indicates whether the architecture is a pure MLP (multi-layer preceptron), includes convolutional layers (CONV), or is a generative model using a DBN (deep belief network) with RBMs (Restricted Boltzmann Machine). Columns “FE Training” and “Classifier Training” specify how the hidden and output classifier layers have been trained. The columns on“Resolution” indicate the resolution used for the weights of the synapses in the hidden and the classifier layers. Column “Classification Accuracy” (CA) compares the recognition performance obtained for the different architectures and methods. We can see that for the systems using some type of synaptic precision reduction (below 8-bit) combined with STDP, the technique presented in this paper shows the best CA for the specific cases analyzed. The only system with weight precision reduction (8-bit) but without STDP that improves our CA is the one by Neftci (Neftci et al., 2017) based on event-driven random backpropagation. For the cases with full precision but using STDP, only (Kheradpisheh et al., 2018) improves our CA, but at the expense of using three convolution layers. Table 4 also includes (under “This Work”) the results we obtained with fixed-random weights, which are very similar to the ones obtained by STDP.

**Table 4:** Comparison of reported results on spiking versions of the static MNIST dataset.

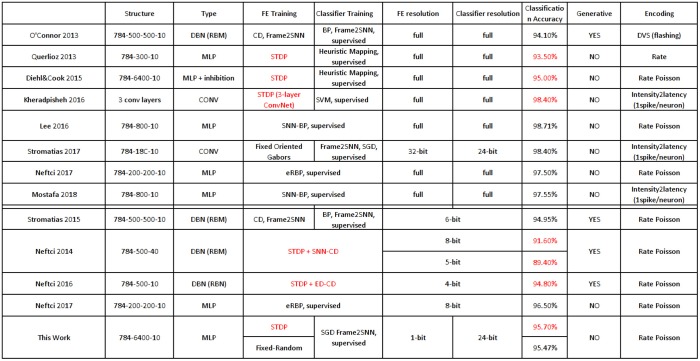

*Experimental results with STDP learning rule are marked in red color*.

The above Tables 3, 4 show that using 1-bit weights for the FE layer (whether fixed-random or trained by STDP) results, from the computational point of view, in overall CAs which are comparable to related state of the art results on limited precision weights and STDP learning systems. However, the most interesting benefits of the presented 1-bit weight technique is its efficiency for hardware implementations. There are not many STDP hardware systems reported, implemented on FPGAs. Table 5 compares similar hardware systems implementing on-line STDP on spiking neural networks. Spartan-3 FPGAs use (according to our experience) about 10% more resources than Spartan-6 for the same system. As we can see, our technique results in about two orders of magnitude in resources consumption (slices per neuron) efficiency with respect to (Rice et al., 2009), and about one order of magnitude with respect to (Cassidy et al., 2007) (and without using Block-RAM). This is achieved thanks to the fact of using 1-bit weights, resulting in high reductions in memory resources, computing resources, and communication resources.

**Table 5:** Comparison of reported results on STDP MLP FPGA Hardware.

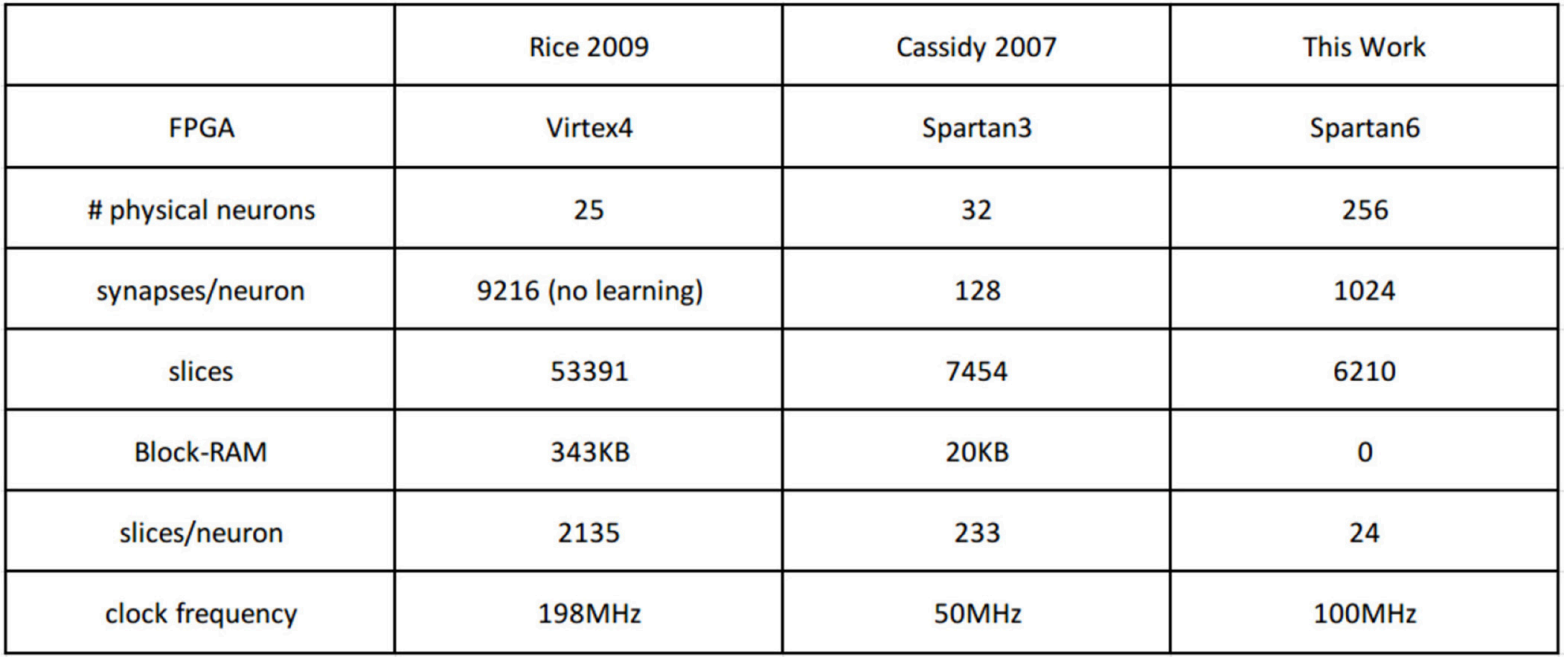

There are some more recent works reporting on STDP based synapses implemented on FPGAs (Pedroni et al., 2016; Jokar and Soleimani, 2017; Nouri et al., 2018; Lammie et al., 2018). Unfortunately, hardware resources are reported only for a single STDP synapse/unit, making it difficult to compare with a full STDP system implementation, since it is not clear how reported synaptic resources can be shared or time-multiplexed at system level. Also, the resources for the rest of computing/memory/communication requirements are not specified.

## Author contributions

ES and AY developed the learning rules, ES worked on the software simulations of orientation selectivity and MNIST, MS on the software simulations of poker-DVS, AY developed all the hardware experiments, TS-G and BL-B supervised the work.

### Conflict of interest statement

The authors declare that the research was conducted in the absence of any commercial or financial relationships that could be construed as a potential conflict of interest.

## Figures and Tables

**Figure A1:**
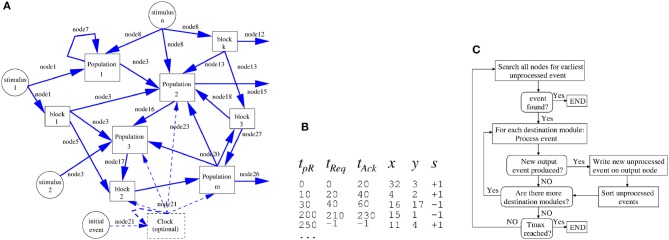
Illustration of generic multi-module/population for MegaSim Event-Driven simulation. **(A)** Nodes and links, **(B)** a section of an event list, **(C)** Megasim's data flow and control diagram.

## Footnotes

^1^The dominant power is typically burnt in the continuous and massive data transfers between computing circuits and memory circuits. If computing and memory circuits are in separate chips, data transfer power is multiplied by one or two orders of magnitude.

^2^Strictly speaking, the correct formulation should say −*T*_*min*_ = 0. This is because for Δ*t* < 0 (pre after post), a new arriving pre-synaptic spike checks the list of prior post-synaptic spikes to perform weight update. This means, that for every pre-synaptic spike, all synapses connecting to this pre-synaptic neuron should also be depressed. However, this action is not taken (Bichler et al., 2012; Querlioz et al., 2013; Roclin et al., 2013; Suri et al., 2013).

^3^Strictly speaking, probabilities are positive numbers. Here we consider that a positive probability provides a probability for potentiation, and a negative probability provides a probability for depression.

^4^Obtaining representative features should make the task of the classifier layer more easy.

^5^In conventional neural networks (with weights of higher resolution) scaling the weights by a factor α is equivalent to scaling the threshold by 1/α (previous “trick”). But here weights are either “0” or “1” and cannot be scaled. It is the total number of weights with value “1” that is kept constant. Also *W*_*sum*_ is the same for all neurons in the population, while in the previous “trick” each neuron can change its threshold separately.

^6^List flushing is similar to implementing lateral inhibition by resetting the neighbors, because neighboring neurons start integrating new events from scratch.

^7^https://bitbucket.org/bernabelinares/megasim.

^8^All HDL codes are available upon request for academic purposes.

^9^It should be clarified that reducing the clock frequency helps to reduce the number of of slices. For lower speeds, fewer resources are required, in general.

^10^Reducing clock frequency helps reducing the number of slices.

^11^Note that for the hardware implementation we have used a more sophisticated leak than for the software implementations. In software we used plain linear leak, while in hardware we are approximating an exponential leak. However, the circuit implementation is simpler: the plain linear leak requires one subtracter and one comparator, while the piece-wise-linear exponential approximation requires one subtracter and bit-shifting.

^12^Spartan-3 FPGA is older than Spartan-6 and, by our experience, a similar design may take around 10% fewer slices in Spartan-6 in comparison to Spartan-3.

^13^A video showing the evolution of the 1-bit weights during stochastic STDP learning can be seen in (Yousefzadeh, 2017a).

^14^We did not consider here MNIST spiking datasets obtained by motion induced recordings with DVS cameras, like N-MNIST (Orchard et al., 2015) or MNIST-DVS (Serrano-Gotarredona and Linares-Barranco, 2015). This will be the subject of future work.

^15^Although our system is not generative, we have included here some generative models that use STDP.

## Appendix

In MegaSim the user can describe any arbitrary topology in a text-based file known as the *netlist* file, where different modules (or populations) are interconnected through AER (Address Event Representation) links, called *nodes*, as shown in Figure A1A. An AER link is a physical high-speed digital multi-bit bus where events are sent at very high speed (typically in the order of a few nano-seconds per event), normally using an asynchronous handshaking protocol with request (Req) and acknowledge (Ack) signals (Serrano-Gotarredona et al., 2009; Zamarreno-Ramos et al., 2013). The modules send events (spikes) through their output ports to the AER links, which are received by the destination modules through their input ports. Both, input and output ports can be shorted among different modules (see for example nodes 3, 8, 20, and 21 in Figure A1A).

For each node there is one event list in a separate file. An event list reports all the events that have been (or will be) communicated through this node (link). If an event has already been processed, it is properly time-stamped. In MegaSim, each event is described by a set of timestamp parameters, and a set of event parameters. Figure A1B shows a section of such an event list. There are 3 timing parameters: *t*_*pR*_ is the time at which an event is “created” inside a module and queued for transfer, *t*_*Req*_ is the time at which this event is physically placed on the intercommunication node, and *t*_*Ack*_ is the time at which this event physically disappears from the intercommunication node, meaning the physical event communication has finished. If *t*_*pR*_ = *t*_*Ack*_ = −1, it means that this event is still waiting to be processed by MegaSim. The AER events used in MegaSim have these three timing values and *n* event parameters. The default event parameters are [*x, y, s*], to signify the (*x, y*) address and the sign or polarity (on/off) of an event^16^.

A system topology like the one shown in Figure A1A is represented by a netlist text file. Each line in the netlist file represents a *module* with its input nodes and output nodes. Each module can be a population of neurons (like a fully connected perceptron layer, a convolution feature map, etc) or any arbitrary event processing algorithm. All modules are described behaviorally in the C programming language. Additionally, each module line includes the names of a separate parameter file and initial state file for this module. Each netlist must contain at least one event *stimulus* node, which can be either data recorded by a physical sensor (like a DVS Lichtsteiner et al., 2008; Posch et al., 2011; Leñero-Bardallo et al., 2011; Serrano-Gotarredona and Linares-Barranco, 2013), or synthetically generated events. At the beginning of a simulation all node files are empty, except for the *stimulus* nodes, which contain all their events but with unprocessed timestamps. Operation is fully asynchronous. However, one can optionally include a clock module that provides an output event after a fixed delay with respect to its input event, and provide a feedback from the output port to the input port, together with a single initial event as stimulus, as shown in Figure A1A.

The simulator data flow and control diagram is depicted in Figure A1C: MegaSim checks all available nodes for the earliest “*unprocessed”* event (*t*_*pR*_ = *t*_*Ack*_ = −1). As soon as it finds one, it marks it as “*processed”* and calls the receiving modules to perform the necessary processing. Should a module generate a new event at one of the output ports, the event will be written on that node with the current time timestamp for *t*_*pR*_ (or larger value, in case a processing delay is modeled) and will be marked as “*unprocessed”*. All unprocessed events are sorted according to their *t*_*pR*_ timestamps. The simulation ends when a maximum simulation time *T*_*max*_ has been reached or when there are no more “*unprocessed”* events. When the simulation finishes, there is a separate event file for each *node* containing its list of events. Each node event list (or combination of lists) can be visualized off-line after the simulation is finished using jAER (Delbruck, 2007), or in real-time while the simulation is running. All MegaSim simulation files are available as supplementary material.

## References

[B1] Barbour B. Brunel N. Hakim V. Nadal J.-P. (2007). What can we learn from synaptic weight distributions? Trends Neurosci. 30, 622–629. 10.1016/j.tins.2007.09.005 17983670

[B2] Bi G. Poo M. (1998). Synaptic modifications in cultured hippocampal neurons: dependence on spike timing, synaptic strength, and postsynaptic cell type. J. Neurosci. 18, 10464–10472. 9852584 10.1523/JNEUROSCI.18-24-10464.1998 PMC6793365

[B3] Bi G. Poo M. M. (2001). Synaptic modification by correlated activity: Hebb's postulate revisited. Ann. Rev. Neurosci. 24, 139–166. 10.1146/annurev.neuro.24.1.139 11283308

[B4] Bichler O. Querlioz D. Thorpe S. J. Bourgoin J.-P. Gamrat C. (2012). Extraction of temporally correlated features from dynamic vision sensors with spike-timing-dependent plasticity. Neural Netw. 32, 339–348. 10.1016/j.neunet.2012.02.022 22386501

[B5] Bienenstock E. Cooper L. Munro P. (1982). Theory for the development of neuron selectivity: orientation specificity and binocular interaction in visual cortex. J. Neurosci. 2, 32–48. 7054394 10.1523/JNEUROSCI.02-01-00032.1982 PMC6564292

[B6] Biswas A. Chandrakasan A. P. (2018). Conv-ram: An energy-efficient sram with embedded convolution computation for low-power cnn-based machine learning applications, in Proceedings of the 2018 International Solid-State Circuits Conference (San Francisco, CA).

[B7] Bong K. Choi S. Kim C. Kang S. Kim Y. (2017). A 0.62mw ultra-low-power convolutional-neural-network face-recognition processor and a cis integrated with always-on haar-like face detector, in Proceedings of the 2017 International Solid-State Circuits Conference (San Francisco, CA), 344–346.

[B8] Bottou L. (2010). Large-Scale Machine Learning with Stochastic Gradient Descent. Heidelberg: Physica-Verlag HD.

[B9] Camuñas-Mesa L. Acosta-Jiménez A. Zamarreno-Ramos C. Serrano-Gotarredona T. Linares-Barranco B. (2011). A 32 x 32 pixel convolution processor chip for address event vision sensors with 155 ns event latency and 20 Meps throughput. IEEE Trans. Circuits Syst. I Regul. Pap. 58, 777–790. 10.1109/TCSI.2010.2078851

[B10] Camuñas-Mesa L. Pérez-Carrasco J. Zamarreño-Ramos C. Serrano-Gotarredona T. Linares-Barranco B. (2010). On scalable spiking convnet hardware for cortex-like visual sensory processing systems, in Circuits and Systems (ISCAS), Proceedings of 2010 IEEE International Symposium on (Paris), 249–252.

[B11] Camuñas-Mesa L. Zamarreno-Ramos C. Linares-Barranco A. Acosta-Jiménez A. J. Serrano-Gotarredona T. Linares-Barranco B. (2012). An event-driven multi-kernel convolution processor module for event-driven vision sensors. IEEE J. Solid State Circuits 47, 504–517. 10.1109/JSSC.2011.2167409

[B12] Cassenaer S. Laurent G. (2006). Hebbian stdp in mushroom bodies facilitates the synchronous flow of olfactory information in locusts. Nature 448, 709–713. 10.1038/nature05973 17581587

[B13] Cassidy A. Denham S. Kanold P. Andreou A. (2007). Fpga based silicon spiking neural array, in 2007 IEEE Biomedical Circuits and Systems Conference (Montréal, QC), 75–78.

[B14] Cassidy A. S. Merolla P. Arthur J. V. Esser S. K. Jackson B. Alvarez-Icaza R. (2013). Cognitive computing building block: a versatile and efficient digital neuron model for neurosynaptic cores, in The 2013 International Joint Conference on Neural Networks (IJCNN) (Dallas, TX), 1–10.

[B15] Chen Y. H. Krishna T. Emer J. (2016). Eyeriss: an energy-efficient reconfigurable accelerator for deep convolutional neural networks, in Proceedings of the 2016 International Solid-State Circuits Conference (San Francisco, CA), 262–263.

[B16] Courbariaux M. Bengio Y. David J.-P. (2015). Binaryconnect: Training deep neural networks with binary weights during propagations, in Advances in Neural Information Processing Systems 28, eds Cortes C. Lawrence N. D. Lee D. D. Sugiyama M. Garnett R. (Curran Associates, Inc.), 3123–3131.

[B17] Dan Y. Poo M. (1998). Hebbian depression of isolated neuromuscular synapse in vitro. Science 256, 1570–1573. 10.1126/science.1317971 1317971

[B18] Davies M. Srinivasa N. Lin T. H. Chinya G. Cao Y. Choday S. H. (2018). Loihi: A neuromorphic manycore processor with on-chip learning. IEEE Micro 38, 82–99. 10.1109/MM.2018.112130359

[B19] Delbruck T. (2007). Real time sensory-motor processing for event-based sensors and systems. Available online at: https://sourceforge.net/p/jaer/wiki/Home/

[B20] Diehl P. Cook M. (2015). Unsupervised learning of digit recognition using spike-timing-dependent plasticity. Front. Comput. Neurosci. 9:99 10.3389/fncom.2015.00099 26941637 PMC4522567

[B21] Diehl P. U. Neil D. Binas J. Cook M. Liu S. C. Pfeiffer M. (2015). Fast-classifying, high-accuracy spiking deep networks through weight and threshold balancing, in 2015 International Joint Conference on Neural Networks (IJCNN) (Killarney), 1–8.

[B22] Feldman D. (2000). Timing-based ltp and ltd at vertical inputs to layer ii/iii pyramidal cells in rat barrel cortex. Neuron 27, 45–56. 10.1016/S0896-6273(00)00008-8 10939330

[B23] Furber S. B. Galluppi F. Temple S. Plana L. A. (2014). The spinnaker project. Proc. IEEE 102, 652–665. 10.1109/JPROC.2014.2304638

[B24] Galluppi F. Lagorce X. Stromatias E. Pfeiffer M. Plana L. A. Furber S. B. (2015). A framework for plasticity implementation on the spinnaker neural architecture. Front. Neurosci. 8:429 10.3389/fnins.2014.00429 25653580 PMC4299433

[B25] Gerstner W. Ritz R. Hemmen J. L. (1993). Why spikes? hebbian learning and retrieval of time-resolved excitation patterns. Biol. Cybern. 69, 503–515. 7903867

[B26] Gonugondla S. K. Kang M. Shanbhag N. (2018). A 42pj/decision 3.12tops/w robust in-memory machine learning classifier with on-chip training, in Proceedings of the 2018 International Solid-State Circuits Conference (San Francisco, CA).

[B27] Guo M. Huang J. Chen S. (2017). Live demonstration: A 768 × 640 pixels 200Meps dynamic vision sensor, in 2017 IEEE International Symposium on Circuits and Systems (ISCAS) (Baltimore, MD), 1.

[B28] Huang G.-B. Zhu Q.-Y. Siew C.-K. (2006). Extreme learning machine: theory and applications. Neurocomputing 70, 489–501. 10.1016/j.neucom.2005.12.126

[B29] Iakymchuk T. Rosado A. Linares-Barranco A. Jiménez-Fernández A. Jiménez-Moreno G. Serrano-Gotarredona T. (2014). An AER Handshake-Less Modular Infrastructure PCB with x8 2.5Gbps LVDS Serial Links, Proceedings of the IEEE International Symposium on Circuits and Systems (Melbourne, VIC), 1556–1559.

[B30] Jacob V. Brasier D. J. Erchova I. Feldman D. Shulz D. E. (2007). Spike-timing-dependent synaptic depression in the *in vivo* barrel cortex of the rat. J. Neurosci. 27, 1271–1284. 10.1523/JNEUROSCI.4264-06.2007 17287502 PMC3070399

[B31] Jeyabalaratnam J. Bharmauria V. Bachatene L. Cattan S. Angers A. Molotchnikoff S. (2013). Adaptation shifts preferred orientation of tuning curve in the mouse visual cortex. PLoS ONE 8:e64294 10.1371/journal.pone.0064294 23717586 PMC3662720

[B32] Jokar E. Soleimani H. (2017). Digital multiplierless realization of a calcium-based plasticity model. IEEE Trans. Circuits Syst. II. Express Briefs 64, 832–836. 10.1109/TCSII.2016.2621823

[B33] Kaiser J. Zimmerer D. Tieck J. C. V. Ulbrich S. Roennau A. Dillmann R. (2017). Spiking convolutional deep belief networks, in Artificial Neural Networks and Machine Learning – ICANN 2017, eds Lintas A. Rovetta S. Verschure P. Villa A. (Cham: Springer International Publishing), 3–11.

[B34] Kheradpisheh S. R. Ganjtabesh M. Thorpe S. J. Masquelier T. (2016). STDP-based spiking deep neural networks for object recognition. CoRR, abs/1611.01421. 10.1016/j.neunet.2017.12.005 29328958

[B35] Kheradpisheh S. R. Ganjtabesh M. Thorpe S. J. Masquelier T. (2018). Stdp-based spiking deep convolutional neural networks for object recognition. Neural Netw. 99, 56–67. 10.1016/j.neunet.2017.12.005 29328958

[B36] Khwa W.-S. Chen J.-J. Li J.-F. Si X. Yang E.-Y. Sun X. (2018). A 65nm 4kb algorithm-dependent computing-in- memory sram unit-macro with 2.3ns and 55.8tops/w fully parallel product-sum operation for binary dnn edge processors, in Proceedings of the 2018 International Solid-State Circuits Conference (San Francisco, CA).

[B37] Lagorce X. Orchard G. Gallupi F. Shi B. E. Benosman R. (2016). HOTS: a Hierarchy Of event-based Time-Surfaces for pattern recognition. IEEE Trans. Pattern Anal. Mach. Intell. 39, 1346–1359. 10.1109/TPAMI.2016.2574707 27411216

[B38] Lammie C. Hamilton T. Rahimi M. (2018). Unsupervised character recognition with a simplified fpga neuromorphic system, in 2018 IEEE International Symposium on Circuits and Systems (ISCAS) (Florence).

[B39] LeCun Y. Bottou L. Bengio Y. Haffner P. (1998). Gradient-based learning applied to document recognition. Proc. IEEE 86, 2278–2324.

[B40] Lee J. H. Delbruck T. Pfeiffer M. (2016). Training deep spiking neural networks using backpropagation. Front. Neurosci. 10:508 10.3389/fnins.2016.00508 PMC5099523 27877107

[B41] Leñero-Bardallo J. A. Serrano-Gotarredona T. Linares-Barranco B. (2011). A 3.6μs latency asynchronous frame-free event-driven dynamic-vision-sensor. IEEE J. Solid State Circuits 46, 1443–1455. 10.1109/JSSC.2011.2118490

[B42] Lichtsteiner P. Posch C. Delbrück T. (2008). A 128 × 128 120dB 30mW asynchronous vision sensor that responds to relative intensity change. IEEE J. Solid State Circuits 43, 566–576. 10.1109/ISSCC.2006.1696265

[B43] Maass W. (2000). On the computational power of winner-Take-All. Neural Comput. 12, 2519–2535. 10.1162/089976600300014827 11110125

[B44] Markram H. Lübke J. Frotscher M. Sakmann B. (1997). Regulation of synaptic efficacy by coincidence of postsynaptic aps and epsps. Science 275, 213–215. 8985014 10.1126/science.275.5297.213

[B45] Masquelier T. Guyonneau R. Thorpe S. J. (2008). Spike timing dependent plasticity finds the start of repeating patterns in continuous spike trains. PLoS ONE 3:e1377 10.1371/journal.pone.0001377 18167538 PMC2147052

[B46] Masquelier T. Guyonneau R. Thorpe S. J. (2009). Competitive stdp-based spike pattern learning. Neural Comput. 21, 1259–1276. 10.1162/neco.2008.06-08-804 19718815

[B47] Masquelier T. and Thorpe, S. (2007). Unsupervised learning of visual features through spike timing dependent plasticity. PLOS Comput. Biol. 3:e31 10.1371/journal.pcbi.0030031 17305422 PMC1797822

[B48] Moore B. IV. Freeman R. (2012). Development of orientation tuning in simple cells of primary visual cortex. J. Neurophysiol. 107, 2506–2516. 10.1152/jn.00719.2011 22323631 PMC3362253

[B49] Mostafa H. (2018). Supervised learning based on temporal coding in spiking neural networks. IEEE Trans. Neural Netw. Learn. Syst. 29, 3227–3235. 10.1109/TNNLS.2017.2726060 28783639

[B50] Mozafari M. Kheradpisheh S. R. Masquelier T. Nowzari-Dalini A. Ganjtabesh M. (2017). First-spike based visual categorization using reward-modulated stdp. arXiv[preprint]arXiv:1705.09132. 10.1109/TNNLS.2018.2826721 29993898

[B51] Mu Y. Poo M. M. (2006). Spike timing-dependent ltp/ltd mediates visual experience-dependent plasticity in a developing retinotectal system. Neuron 50, 115–125. 10.1016/j.neuron.2006.03.009 16600860

[B52] Neftci E. Das S. Pedroni B. Kreutz-Delgado K. Cauwenberghs G. (2015). Event-driven contrastive divergence: neural sampling foundations. Front. Neurosci. 9:104 10.3389/fnins.2015.00104 25873857 PMC4379871

[B53] Neftci E. O. Augustine C. Paul S. Detorakis G. (2017). Event-driven random back-propagation: enabling neuromorphic deep learning machines. Front. Neurosci. 11:324 10.3389/fnins.2017.00324 PMC5478701 28680387

[B54] Neftci E. O. Pedroni B. U. Joshi S. Al-Shedivat M. Cauwenberghs G. (2016). Stochastic synapses enable efficient brain-inspired learning machines. Front. Neurosci. 10:241 10.3389/fnins.2016.00241 PMC4925698 27445650

[B55] Negri P. (2018). Shapes characterization on address event representation using histograms of oriented events and an extended LBP approach. CoRR, arXiv[Preprint]arXiv:1802.03327.

[B56] Nouri M. Jalilian M. Hayati M. Abbott D. (2018). A digital neuromorphic realization of pair-based and triplet-based spike-timing-dependent synaptic plasticity. IEEE Trans. Circuits Syst. II Express Briefs 65, 804–808. 10.1109/TCSII.2017.2750214

[B57] Nowotny T. (2014). Two challenges of correct validation in pattern recognition. Front. Robot. AI 1:5 10.3389/frobt.2014.00005

[B58] O'Connor P. Neil D. Liu S.-C. Delbruck T. Pfeiffer M. (2013). Real-time classification and sensor fusion with a spiking deep belief network. Front. Neurosci. 7:178 10.3389/fnins.2013.00178 24115919 PMC3792559

[B59] Orchard G. Meyer C. Etienne-Cummings R. Posch C. Thakor N. Benosman R. (2015). HFirst: a temporal approach to object recognition. IEEE Trans. Pattern Anal. Mach. Intell. 37, 2028–2040. 10.1109/TPAMI.2015.2392947 26353184

[B60] Pedroni B. U. Sheik S. Joshi S. Detorakis G. Paul S. Augustine C. (2016). Forward table-based presynaptic event-triggered spike-timing-dependent plasticity, in 2016 IEEE Biomedical Circuits and Systems Conference (BioCAS) (Shanghai), 580–583.

[B61] Pérez-Carrasco J. A. Zhao B. Serrano C. Acha B. Serrano-Gotarredona T. Chen S. Linares-Barranco B. (2013). Mapping from frame-driven to frame-free event-driven vision systems by low-rate rate coding and coincidence processing. Application to feedforward convNets. IEEE Trans. Pattern Anal. Mach. Intell. 35, 2706–2719. 10.1109/TPAMI.2013.71 24051730

[B62] Posch C. Matolin D. Wohlgenannt R. (2011). A QVGA 143 dB dynamic range frame-free PWM image sensor with lossless pixel-level video compression and time-domain CDS. IEEE J. Solid State Circuits 46, 259–275. 10.1109/JSSC.2010.2085952

[B63] Querlioz D. Bichler O. Dollfus P. Gamrat C. (2013). Immunity to device variations in a spiking neural network with memristive nanodevices. IEEE Trans. Nanotechnol. 12, 288–295. 10.1109/TNANO.2013.2250995

[B64] Rastegari M. Ordonez V. Redmon J. Farhadi A. (2016). Xnor-net: Imagenet classification using binary convolutional neural networks, in European Conference on Computer Vision (Amsterdam: Springer), 525–542.

[B65] Rice K. L. Bhuiyan M. A. Taha T. M. Vutsinas C. N. Smith M. C. (2009). Fpga implementation of izhikevich spiking neural networks for character recognition, in 2009 International Conference on Reconfigurable Computing and FPGAs (Quintana Roo), 451–456.

[B66] Roberts P. D. Bell C. C. (2002). Spike timing dependent synaptic plasticity in biological systems. Biol. Cybern. 87, 392–403. 10.1007/s00422-002-0361-y 12461629

[B67] Roclin D. Bichler O. Gamrat C. Thorpe S. J. Klein J. O. (2013). Design study of efficient digital order-based STDP neuron implementations for extracting temporal features, in The 2013 International Joint Conference on Neural Networks (IJCNN) (Dallas, TX), 1–7.

[B68] Seo J.-S. Seok M. (2015). Digital cmos neuromorphic processor design featuring unsupervised online learning, in 2015 IFIP/IEEE International Conference on Very Large Scale Integration (VLSI-SoC) (Daejeon: IEEE), 49–51.

[B69] Serrano-Gotarredona R. Öster M. Lichtsteiner P. Linares-Barranco A. Paz-Vicente R. Gómez-Rodríguez F. (2009). CAVIAR: A 45k neuron, 5M synapse, 12G connect/s AER hardware sensory-processing-learning-actuating system for high speed visual object recognition and tracking. IEEE Trans. Neural Netw. 20, 1417–1438. 10.1109/TNN.2009.2023653 19635693

[B70] Serrano-Gotarredona T. Linares-Barranco B. (2013). A 128 × 128 1.5% contrast sensitivity 0.9% FPN 3 μs latency 4 mW asynchronous frame-free dynamic vision sensor using transimpedance preamplifiers. IEEE J. Solid State Circuits 48, 827–838. 10.1109/JSSC.2012.2230553

[B71] Serrano-Gotarredona T. Linares-Barranco B. (2015). Poker-DVS and MNIST-DVS. their history, how they were made, and other details. Front. Neurosci. 9:481 10.3389/fnins.2015.00481 26733794 PMC4686704

[B72] Serrano-Gotarredona T. Linares-Barranco B. Galluppi F. Plana L. Furber S. (2015). ConvNets experiments on SpiNNaker, in 2015 IEEE International Symposium on Circuits and Systems (ISCAS), 2405–2408.

[B73] Sim J. Park J. Kim M. Bae D. Choi Y. (2016). A 1.42tops/w deep convolutional neural network recognition processor for intelligent ioe systems, in Proceedings of the 2016 International Solid-State Circuits Conference (San Francisco, CA), 264–265.

[B74] Son B. Suh Y. Kim S. Jung H. Kim J. S. Shin C. (2017). 4.1 A 640x480 dynamic vision sensor with a 9um pixel and 300Meps address-event representation, in 2017 IEEE International Solid-State Circuits Conference (ISSCC) (San Francisco, CA), 66–67.

[B75] Soto M. (2017). Slow Poker DVS Data Set. Available online at: http://www2.imse-cnm.csic.es/caviar/SLOWPOKERDVS.html

[B76] Stromatias E. Neil D. Pfeiffer M. Galluppi F. Furber S. B. Liu S.-C. (2015). Robustness of spiking deep belief networks to noise and reduced bit precision of neuro-inspired hardware platforms. Front. Neurosci. 9:222 10.3389/fnins.2015.00222 26217169 PMC4496577

[B77] Stromatias E. Soto M. Serrano-Gotarredona T. Linares-Barranco B. (2017). An event-driven classifier for spiking neural networks fed with synthetic or dynamic vision sensor data. Front. Neurosci. 11:350 10.3389/fnins.2017.00350 PMC5487436 28701911

[B78] Suri M. Querlioz D. Bichler O. Palma G. Vianello E. Vuillaume D. (2013). Bio-inspired stochastic computing using binary cbram synapses. IEEE Trans. Electron Devices 60, 2402–2409. 10.1109/TED.2013.2263000

[B79] Thorpe S. Gautrais J. (1998). Rank order coding, in Computational Neuroscience (Boston, MA:Springer US), 113–118.

[B80] Whatmough P. Lee S. Lee H. Rama S. (2017). A 28nm soc with a 1.2ghz 568nj/prediction sparse deep-neural-network engine with >0.1 timing error rate tolerance for iot applications, in Proceedings of the 2017 International Solid-State Circuits Conference (San Francisco, CA), 242–243.

[B81] Yousefzadeh A. (2017a). Real Time Demo, Binary STDP Neurons Learns Poker Card Symbols. Available online at: https://youtu.be/Gv2iVvoLL6A

[B82] Yousefzadeh A. (2017b). Real Time Demo, Binary STDP Online Learning in FPGA for Rotating Bar. Available online at: https://youtu.be/iH6VJl70X5s

[B83] Yousefzadeh A. Masquelier T. Serrano-Gotarredona T. Linares-Barranco B. (2017). Hardware implementation of convolutional STDP for on-line visual feature learning, in 2017 IEEE International Symposium on Circuits and Systems (ISCAS) (Baltimore, MD), 1–4.

[B84] Zamarreno-Ramos C. Linares-Barranco A. Serrano-Gotarredona T. Linares-Barranco B. (2013). Multicasting mesh aer: a scalable assembly approach for reconfigurable neuromorphic structured aer systems. application to convnets. IEEE Trans. Biomed. Circuits Syst. 7, 82–102. 10.1109/TBCAS.2012.2195725 23853282

[B85] Zhang L. Tao H. Holt C. Harris W. Poo M. (1998). A critical window for cooperation and competition among developing retinotectal synapses. Nature 395, 37–44. 9738497 10.1038/25665

